# Long-term prognosis and educational determinants of brain network decline in older adult individuals

**DOI:** 10.1038/s43587-021-00125-4

**Published:** 2021-11-11

**Authors:** Micaela Y. Chan, Liang Han, Claudia A. Carreno, Ziwei Zhang, Rebekah M. Rodriguez, Megan LaRose, Jason Hassenstab, Gagan S. Wig

**Affiliations:** 1 Center for Vital Longevity and School of Behavioral and Brain Sciences, University of Texas at Dallas, Dallas, TX, USA.; 2 Department of Neurology, Washington University School of Medicine, St. Louis, MO, USA.; 3 Department of Psychiatry, University of Texas Southwestern Medical Center, Dallas, TX, USA.

## Abstract

Older adults with lower education are at greater risk for dementia. It is unclear which brain changes lead to these outcomes. Longitudinal imaging-based measures of brain structure and function were examined in adult individuals (baseline age, 45–86 years; two to five visits per participant over 1–9 years). College degree completion differentiates individual-based and neighborhood-based measures of socioeconomic status and disadvantage. Older adults (~65 years and over) without a college degree exhibit a pattern of declining large-scale functional brain network organization (resting-state system segregation) that is less evident in their college-educated peers. Declining brain system segregation predicts impending changes in dementia severity, measured up to 10 years past the last scan date. The prognostic value of brain network change is independent of Alzheimer’s disease (AD)-related genetic risk (*APOE* status), the presence of AD-associated pathology (cerebrospinal fluid phosphorylated tau, cortical amyloid) and cortical thinning. These results demonstrate that the trajectory of an individual’s brain network organization varies in relation to their educational attainment and, more broadly, is a unique indicator of individual brain health during older age.

Educational attainment differences are closely linked to health disparities across individuals (for example, ref.^1^). Adults with higher education live longer and healthier lives than their peers with less education^2^. Conversely, lower education is associated with increased risk of mental health disorders^3,4^ and dementia^5–7^ during advanced age. Education-related differences in health outcomes during older age are likely mediated by a complex combination of socioeconomic factors that are realized via the opportunities that higher education affords over an individual’s adulthood. These factors include access to resources and environmental stimulation, health habits and exposure to different levels and types of stress (for example, ref.^8^; reviewed in ref.^9^). Critically, however, efforts to link an individual’s education and environment to their brain changes, including both brain structure (for example, refs.^10,11^; also see ref.^12^ for review) and measures of brain pathology (for example, refs.^13–15^), have yielded mixed results. Establishing a link between educational attainment and specific brain changes during older age is not only an important step toward understanding environmental determinants of brain disease^16^ but could also catalyze discovery and incorporation of new brain health ‘biomarkers’ (for example, ref.^17^).

Due to its devastating threat to older adults and to public health systems, there is an urgent need to elucidate the causes of AD. Except in rarer forms of the disease (for example, autosomal-dominant AD), there are no known direct determinants of AD, indicating an interaction between genetic risk and various environmental, psychosocial and lifestyle factors^18^. AD brains are characterized by the presence of two types of pathology: abnormal levels of extracellular beta amyloid (Aβ) plaques and intracellular tau proteins in the form of neurofibrillary tangles^19,20^. A recently proposed framework incorporates biomarkers sensitive to both of these neuropathologies (measured using positron emission tomography (PET) or from cerebrospinal fluid (CSF)) as well as measures of neurodegeneration (for example, brain atrophy, assessed by gray matter cortical thinning and volume loss, and hypometabolism, assessed by fluorodeoxyglucose uptake) to help classify and stage AD (that is, the amyloid, tau and neurodegeneration (A/T/N) framework^21^). There has been substantial progress in understanding the trajectory of these AD-related biomarkers (for example, refs.^22–24^) and their potential relationships with impending cognitive decline (for example, refs.^25,26^). However, it has also been clear that individuals with comparable biomarker profiles may still have different clinical profiles^17,27^, suggesting that other moderators exist that have yet to be accounted for, which could also be more broadly informative in understanding trajectories of brain aging.

Progress in incorporating measures of brain function into models of aging, AD and dementia more generally has been slow. This is largely due to inherent constraints associated with characterizing brain signals in older and cognitively impaired populations with task-related functional imaging (that is, challenges with participant compliance and feasibility), but is also due to the complexities of accounting for brain variability associated with differing behavioral performance. However, brain function is also reflected in the correlation structure of brain region signals in the absence of overt task performance (that is, during the ‘resting state’ (ref.^28^)). Resting-state functional correlations (RSFCs) represent ‘Hebbian-like’ statistical histories of coactivation between areas of the brain^29^. When sets of RSFC signals sampled across multiple brain areas are examined in aggregate, complex large-scale brain network organization is evident (review in ref.^30^). While RSFC networks remain relatively stable on a day-to-day basis and with variations of state (for example, ref.^31^), they have been shown to differ across the lifespan (for example, refs.^32–34^), reflecting protracted periods of changes in brain function that accompany childhood development and adult aging. Importantly, RSFC networks vary in relation to cognitive ability among healthy adults (for example, refs.^35,36^) and also differ based on disease status (for example, refs.^37,38^). The present work is motivated by the hypothesis that an individual’s large-scale brain network organization reflects their brain’s functional integrity and that functional brain network degradation may be prognostic of cognitive impairment beyond global measures of brain atrophy and pathological burden.

Large-scale RSFC networks are organized into a modular architecture (for example, ref.^39^); modules correspond to functionally specialized brain systems, and the segregation of these systems supports brain function^40,41^. Multiple reports have now demonstrated that increasing adult age is associated with less-segregated brain networks^33,34,42^. This ‘dedifferentiation’ of functional systems has been associated with age-accompanied differences in patterns of brain activity, worse cognitive and motor ability, lower energy metabolism and altered neurotransmitter levels (for example, refs.^43–47^). Cross-sectional comparisons have also revealed that middle-aged adults with lower socioeconomic status (SES) (35–64 years of age) exhibit lower brain system segregation than that of their peers of the same age with higher SES^48^, suggesting that there may exist environmental determinants of brain network aging. Finally, comparisons of patients with AD relative to age-matched healthier adults have provided some evidence that patterns of reduced segregation exist in the brain’s functional systems among individuals with dementia (for example, refs.^49,50^). This collective work motivates the current study, which aims to determine (1) whether a link exists between an individual’s educational attainment and longitudinal changes in their brain system segregation during adulthood and (2) whether changes in brain network organization are prognostic of impending clinical decline in aging adult individuals.

To answer these questions, we have assembled and examined data from a diverse participant cohort of varying adult ages across multiple longitudinal magnetic resonance imaging (MRI) sessions and clinical visits (including clinical visits that were conducted up to 10 years after the participant’s last available MRI session). The richness of the dataset allows us to examine the relationship between educational attainment and changes in brain system segregation in individual adult participants over time while accounting for demographics and various measures of health and pathology. These brain network changes are then evaluated in relation to trajectories of clinical decline to examine the prognostic utility of changes in functional brain network organization over and above an individual’s AD-related genetic risk and presence of AD-related brain pathology.

## Results

**Fig. 1:**
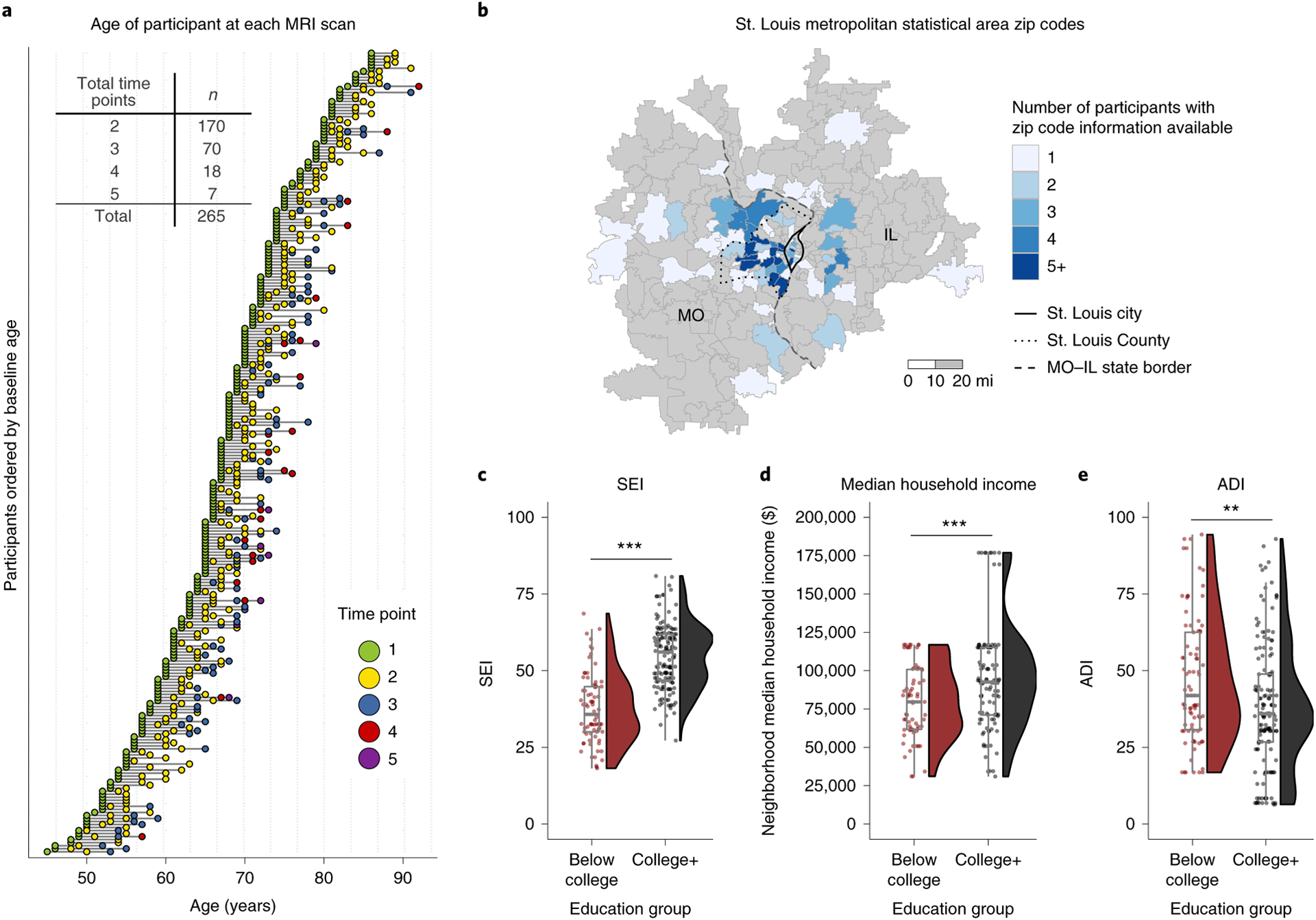
Longitudinal MRI sessions and geographical and SES-related information of participants. **a**, Every participant had at least two MRI (including resting-state) scan sessions, with 36% of participants having three to five separate sessions over multiple years (maximum range, 9.2 years). **b**, Number of participants from zip codes within the St. Louis metropolitan statistical area. The border of the city of St. Louis (solid), the county of St. Louis (dotted) and the state border between Missouri (MO) and Illinois (IL; dashed) are also depicted. **c**, Participants with a college degree have higher socioeconomic index (SEI) (data are available for 86% (*n* = 228) of participants; *t*_132_ = −10.791, *P* < 0.001, Cohen’s *d* = 1.552, 95% confidence interval (CI_95%_) = (−20.122, −13.888)) and **d**, live in areas (calculated using participants’ zip codes) with higher neighborhood median household income (data available for 65% (*n* = 171) of participants; *t*_168_ = −3.455, *P* < 0.001, *d* = −0.520, CI_95%_ (−24,169.742, −6,591.3510); see text for additional comments about outlier observations) and **e**, lower scores on the area deprivation index (ADI), an index that combines multiple environmental variables to characterize area deprivation (data available for 89% (*n* = 236) of participants; *t*_170_ = 3.197, *P* = 0.002, *d* = 0.435, CI_95%_ = (3.417, 14.443)). Each box plot depicts the median (center line) and first and third quartiles (bounds of box), and whiskers extend to 1.5 times the interquartile range. The whisker does not extend to the minimum and maximum of the data, as individual data points are also plotted. Two-sided Welch’s two-sample *t*-tests were performed for **c**–**e** (significance is denoted in **c**–**e** with asterisks: **P < 0.01, ***P < 0.001).

Longitudinal changes in brain system segregation were examined in predominantly cognitively normal middle-aged and older adult participants (*n* = 265; 45–86 years of age at baseline) enrolled in ongoing studies of normal aging and dementia at the Charles F. and Joanne Knight Alzheimer Disease Research Center (Knight ADRC) at Washington University in St. Louis. Each participant had two to five visits during which MRI was conducted, which included resting-state scans, collected over 323–3,372 d (0.88–9.24 years). Over 95% of longitudinal sessions were collected >1 year after previous sessions; all available sessions were included to maximize accuracy of individual change estimates (Fig. 1a). While a large portion of participants in the final sample came from the city of St. Louis and areas in the immediately surrounding St. Louis County, a sizable portion of participants came from other zip codes within the greater St. Louis metropolitan statistical area, which altogether encompassed reasonable geographical diversity (Fig. 1b).

### College degree attainment differentiates individual-based and neighborhood-based measures of socioeconomic advantages

For each individual, educational attainment was considered categorically. The categorical distinction of having a college degree or not has been shown to confer significant health^1,4^ and socioeconomic advantages^51^ even after accounting for the economic costs to obtain the degree. Consistent with this, examination of the present study’s participant characteristics confirmed the relation between education and other socioeconomic outcomes. For participants for whom relevant information was available, a college education (‘college+’ versus ‘below college’) was associated with higher occupation-based socioeconomic index (SEI)^52^ (*t*_132_ = −10.791, *P* < 0.0001, CI_95%_ = (−20.122, −13.888)) (Fig. 1c; CI_95%_ of mean difference is reported), living in neighborhoods that had a higher median household income (*t*_168_ = −3.455, *P* < 0.001, CI_95%_ = (−24,169.742, −6,591.351)) (Fig. 1d; after removing the ten participants that resided in two zip codes with a median household income above $150,000 per year (Ladue, MO and Chesterfield, MO), the estimated median household income remained marginally higher for ‘college+’ participants than that for ‘below college’ participants (*t*_141_ = −1.813, *P* = 0.072, CI_95%_ = (−14,283.871, 618.663)); after outliers were removed, ‘below college’ mean = $79,620.19 and ‘college+’ mean = $86,452.80) and lower scores on an index that combines multiple environmental variables to characterize area deprivation (area deprivation index (ADI)^53^) (*t*_170_ = 3.197, *P* = 0.002, CI_95%_ = (3.417, 14.443)) (a single statistical outlier was present in the ADI data, but removing it did not yield a qualitative difference in the statistical comparison; Fig. 1e).

Participants’ ages at their baseline scan session did not significantly differ between education groups; there was a higher proportion of females in the ‘below college’ group than the ‘college+’ group. Further, with the exception of mild depressive symptoms (measured by the Geriatric Depression Scale (GDS)), variables related to clinical status, AD-related pathology, cardiovascular health and history of traumatic brain injury (TBI) did not differ across education groups. We note that the participant sample was constructed from studies collected at the Knight ADRC, which typically have a higher proportion of participants with the *APOE* ε4 genotype than the general population; however, the proportion of participants positive for the *APOE* ε4 genotype did not significantly differ across education groups (see Table 1 for statistical descriptions and comparisons).

### Older adults with less education exhibit greater declines in brain system segregation

**Fig. 2:**
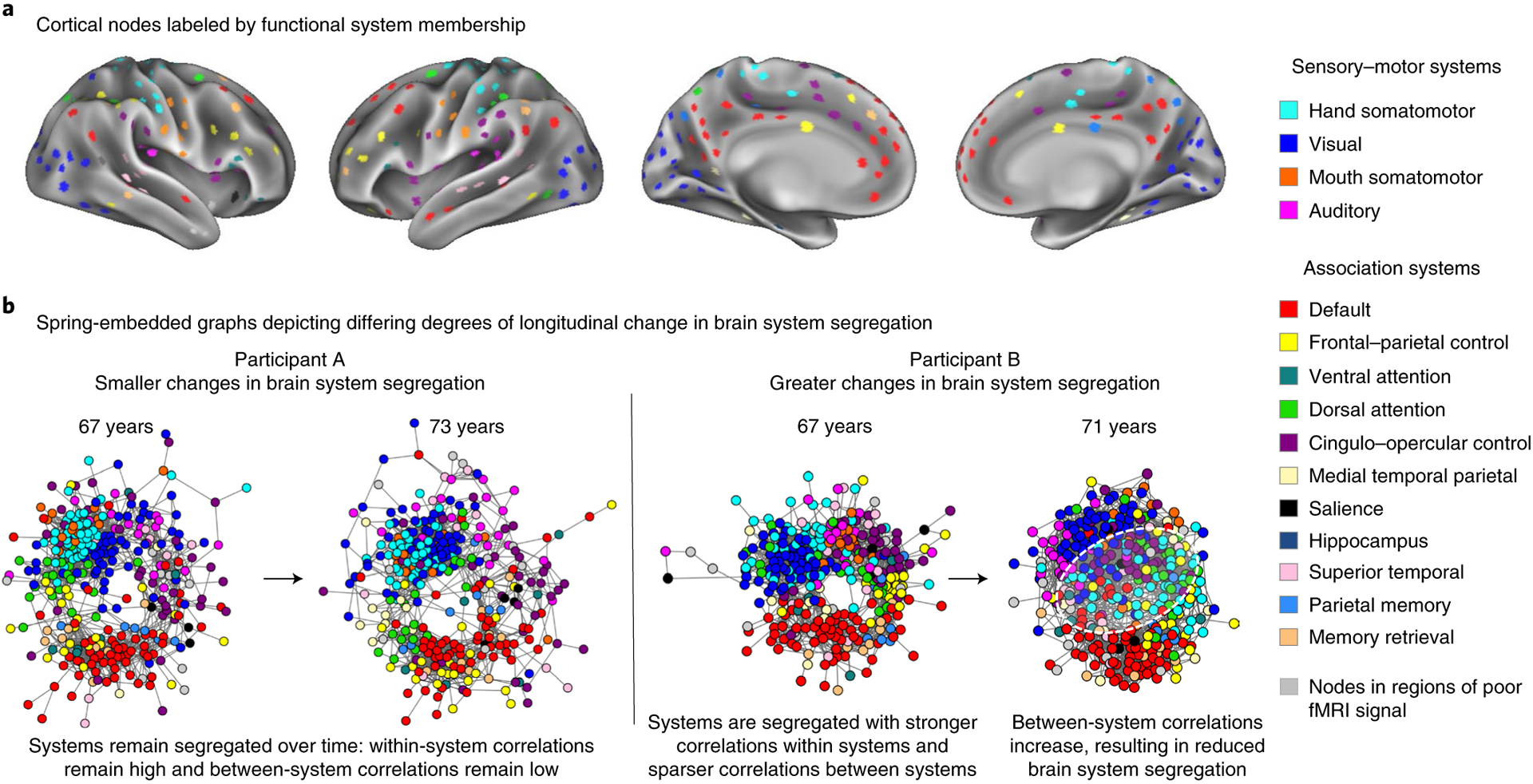
Examining individual changes in resting-state brain system segregation across years. **a**, For each participant, resting-state brain network graphs were created using functional nodes (Chan et al.^33^) labeled by their corresponding functional systems (Power et al.^39^). **b**, While the exact topology of networks differs between individuals, examples of brain graphs are depicted here for two individuals of equivalent age at their first scan but with differing degrees of longitudinal change in brain system segregation. Relative to their baseline networks, greater reductions in brain system segregation are observable in the participant on the right than in the participant on the left over a comparable time span (white dashed circle, reduced separation of brain systems).

Following rigorous quality control and data-cleaning procedures (Methods), each participant’s resting-state functional brain network (brain network graph) was constructed using a surface-based node set (brain area parcellation^33^), with each node labeled according to its functional system assignment^39^ (Fig. 2a). Generally, resting-state correlations between functional areas from the same systems are higher than those of different systems, reflecting a modular network organization (Fig. 2b). This modular organization promotes the functional specialization of distinct systems^41^, which can be effectively summarized using a measure of brain system segregation^33^.

**Fig. 3:**
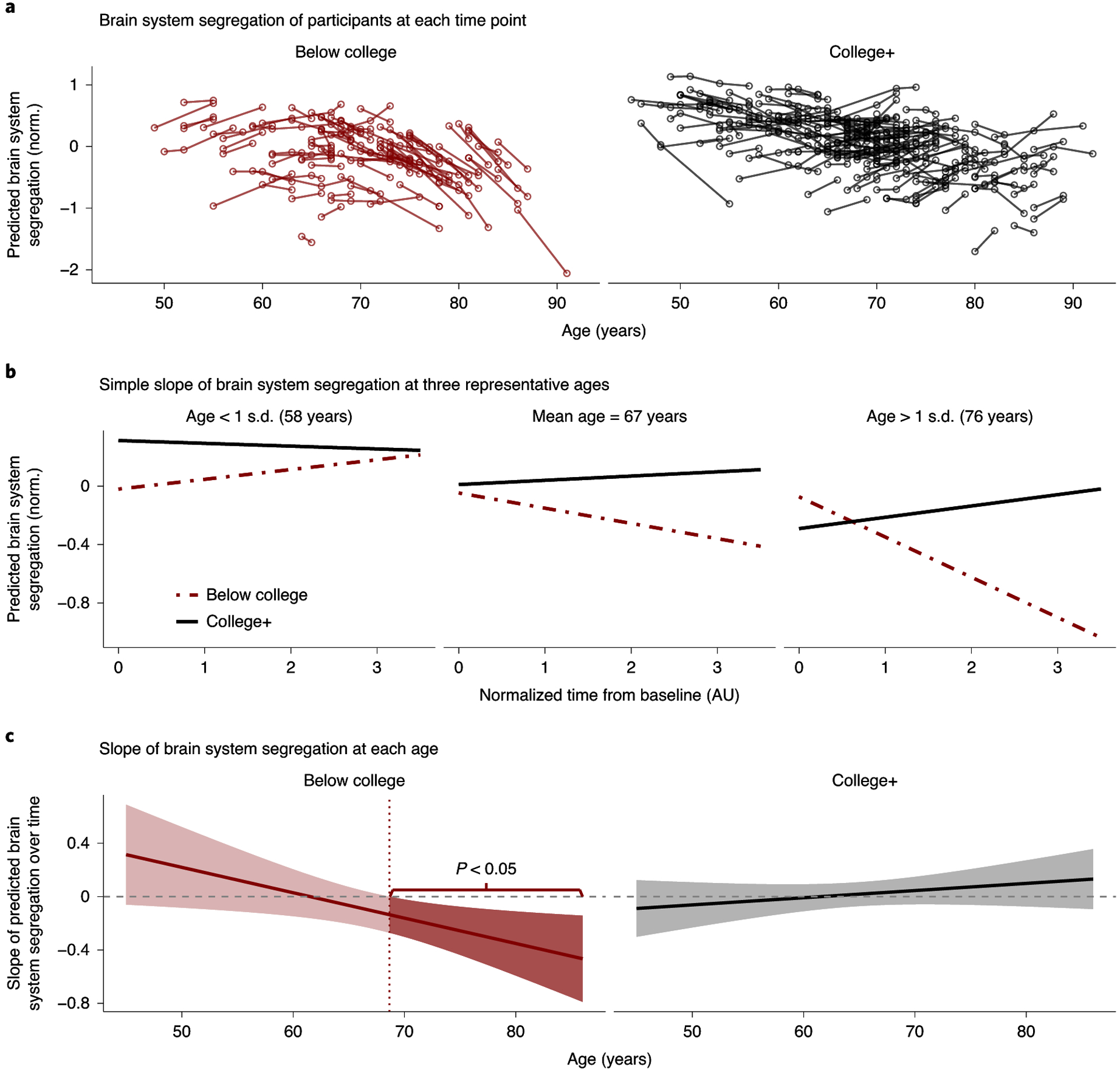
Brain system segregation declines most in older adults without a college degree. **a**, Predicted brain system-segregation values from the linear mixed-effects model plotted against a participant’s age when the brain scan was acquired. A greater decline in brain system segregation is evident in participants who are older (~65 years and over) and without a college degree than in more educated individuals. Norm., normalized. **b**, Simple slope analysis depicts the predicted slope of brain system segregation at three representative ages: 58, 67 and 76 years (age < 1 s.d., mean age and age > 1 s.d. across the entire sample, respectively). Brain system segregation declines in older participants without a college degree (dashed red line, middle and right (ages 67 and 76 years) but stays relatively flat for individuals with a college degree regardless of age (solid black lines). AU, arbitrary units. **c**, A Johnson–Neyman analysis fully illustrates how changes in brain system segregation over time (*y* axis) differ based on the participant’s age (*x* axis) and educational attainment. The predicted slope calculated from the linear mixed-effects model for each age within both education groups is illustrated; envelopes represent CI_95%_. In participants without a college degree (left), the analysis identifies the ages at which longitudinal changes in brain system segregation are statistically significant (darker shade represents a slope with *P* < 0.05, where the CI_95%_ of the slope does not cross 0). The decline in brain system segregation is statistically significant for individuals without a college education starting at approximately 69 years of age; reliable changes in brain system segregation are not evident at any segment of age for those with a college degree (right). To facilitate the comparison between both education groups, the slopes of predicted brain system segregation for both ‘below college’ and ‘college+’ groups are shown for the full age range of the entire sample (45–86 years).

Changes in brain system segregation were examined as a function of participants’ educational attainment and age, with follow-up analyses examining whether this relationship was independent of other health indicators that have established and hypothesized associations with education and brain function. A linear mixed-effects model revealed a significant main effect of education group (*F*_1,271_ = 5.073, *P* = 0.025, CI_95%_ = (−1.973, −0.149); CI_95%_ of the linear mixed-model term estimate is reported) and age at baseline (*F*_1,269_ = 7.029, *P* = 0.008, CI_95%_ = (−0.032, −0.005)); participants with a college degree exhibited a higher level of brain system segregation, and older age was associated with lower brain system segregation. The statistical test also revealed significant interactions between education group and age at baseline (*F*_1,270_ = 4.949, *P* = 0.027, CI_95%_ = (0.002, 0.029)) and between time (that is, normalized from baseline) and education group (*F*_1,186_ = 5.756, *P* = 0.017, CI_95%_ = (0.139, 1.368)). Most importantly, the model revealed a significant three-way interaction between time, education group and age at baseline (*F*_1,193_ = 6.814, *P* = 0.010, CI_95%_ = (−0.021, −0.003)) after accounting for the effect of self-reported sex and in-scanner head motion on brain system segregation. The nature of this interaction can be appreciated in Fig. 3a; older adults (more than ~65 years of age) without a college degree exhibited reliable and declining brain system segregation over time, which was not uniformly evident in older adults with a college degree. These observations were reinforced in simple slope (Fig. 3b) and Johnson–Neyman (Fig. 3c) analyses. In the Johnson–Neyman analysis, it was evident that the predicted slopes in ‘below college’ adults became negative in older age, but the predicted slopes in ‘college+’ adults remained relatively close to zero (that is, a flatter slope). In keeping with this, while the model indicated positive predicted slopes for some individuals in both education groups (for example, younger age for ‘below college’ and older age for ‘college+’), these positive slopes were relatively weaker and not statistically significant, as evidenced by the fact that the confidence intervals for these portions of participants included zero (shaded intervals in Fig. 3c).

Although the proportion of minority (non-white) participants in the present sample is limited (*n* = 26, ~10%), we reanalyzed the model while accounting for participant self-reported race. The three-way interaction between time, education group and age at baseline remained significant for predicting brain system segregation (*F*_1,192_ = 6.399, *P* = 0.012, CI_95%_ = (−0.021, −0.002)). Further, while the proportion of female participants across education groups differed (Table 2), including sex as an interaction term revealed no significant four-way interaction between time, education group, age at baseline and sex (*F*_1,175_ = 0.073, *P* = 0.788, CI_95%_ = (−0.014, 0.010)).

### Education-related brain network decline is independent of clinical status, AD-related genetic risk and pathology, and general measures of health

The participants in the present dataset were recruited in research studies targeting populations with higher AD risk (for example, based on their age, family history or clinical status). Given that lower educational attainment is associated with greater risk of dementia in older age^5^, it is important to determine whether the observed brain network changes are linked to baseline differences in clinical status and/or subclinical AD-related pathology but also other available measures of health more broadly.

Multiple statistical models relating educational attainment to brain system-segregation changes were conducted while accounting for individual variability in measures related to baseline clinical status (clinical dementia rating (CDR);^54^ see section 1.1 in the Supplementary Information for an analysis excluding a small subset of individuals (*n* = 25) with a CDR of 0.5 at baseline, indicating very mild dementia), AD genetic risk (*APOE* status), baseline AD-related pathology (a categorical measure based on the presence of elevated CSF phosphorylated tau 181 (CSF pTau) and/or elevated cortical amyloid levels measured using Pittsburgh compound B (PiB) PET), baseline cardiovascular health (an aggregate measure including body mass index (BMI), incidents of hypertension, hypercholesterolemia and other cardiovascular incidents), baseline depressive symptoms (as measured by the GDS) and history of traumatic brain injury. The results of statistical models incorporating these measures are summarized in Table 2. The observed relationships between educational attainment and changes in brain system segregation largely persisted after accounting for the various measures individually or in aggregate.

### Education-related changes in brain system segregation are not captured by measures of cortical thinning

Multiple studies have reported cross-sectional relationships between environmental variables and brain structure in adulthood (for example, refs.^10,48,55,56^). Accordingly, it is important to determine whether the observed education-related brain network changes are captured by changes in brain structure. We first determined that there was a lack of relationship between brain system segregation and mean cortical thickness after controlling for age-related variance (partial correlation controlling for age, *r*_262_ = 0.066, *P* = 0.284, CI_95%_ = (−0.055, 0.185); raw cross-sectional correlation, *r*_263_ = 0.176, *P* = 0.004, CI_95%_ = (0.057, 0.291)). In keeping with this, the interaction between time, education group and age at baseline predicting brain system segregation remained significant after controlling for longitudinal measures of mean cortical thickness (*F*_1,195_ = 5.915, *P* = 0.016, CI_95%_ = (−0.021, −0.002)).

Modeling longitudinal mean cortical thickness as a dependent measure resulted in a main effect of age at baseline (*F*_1,264_ = 82.683, *P* < 0.001, CI_95%_ = (−0.062, −0.040)) and an interaction between time and age at baseline (*F*_1,173_ = 6.711, *P* = 0.010, CI_95%_ = (−0.011, −0.002)). However, time, age and education group did not significantly interact (*F*_1,178_ = 0.003, *P* = 0.954, CI_95%_ = (−0.005, 0.005)). This observation reveals that longitudinal decline in brain structure is more uniformly observed across participants, and, unlike brain system segregation, it does not vary in relation to educational attainment (see section 1.2 in the Supplementary Information and Supplementary Fig. 1 for simple slope and Johnson–Neyman analyses).

### Aging-related system-specific changes vary as a function of educational attainment

**Fig. 4:**
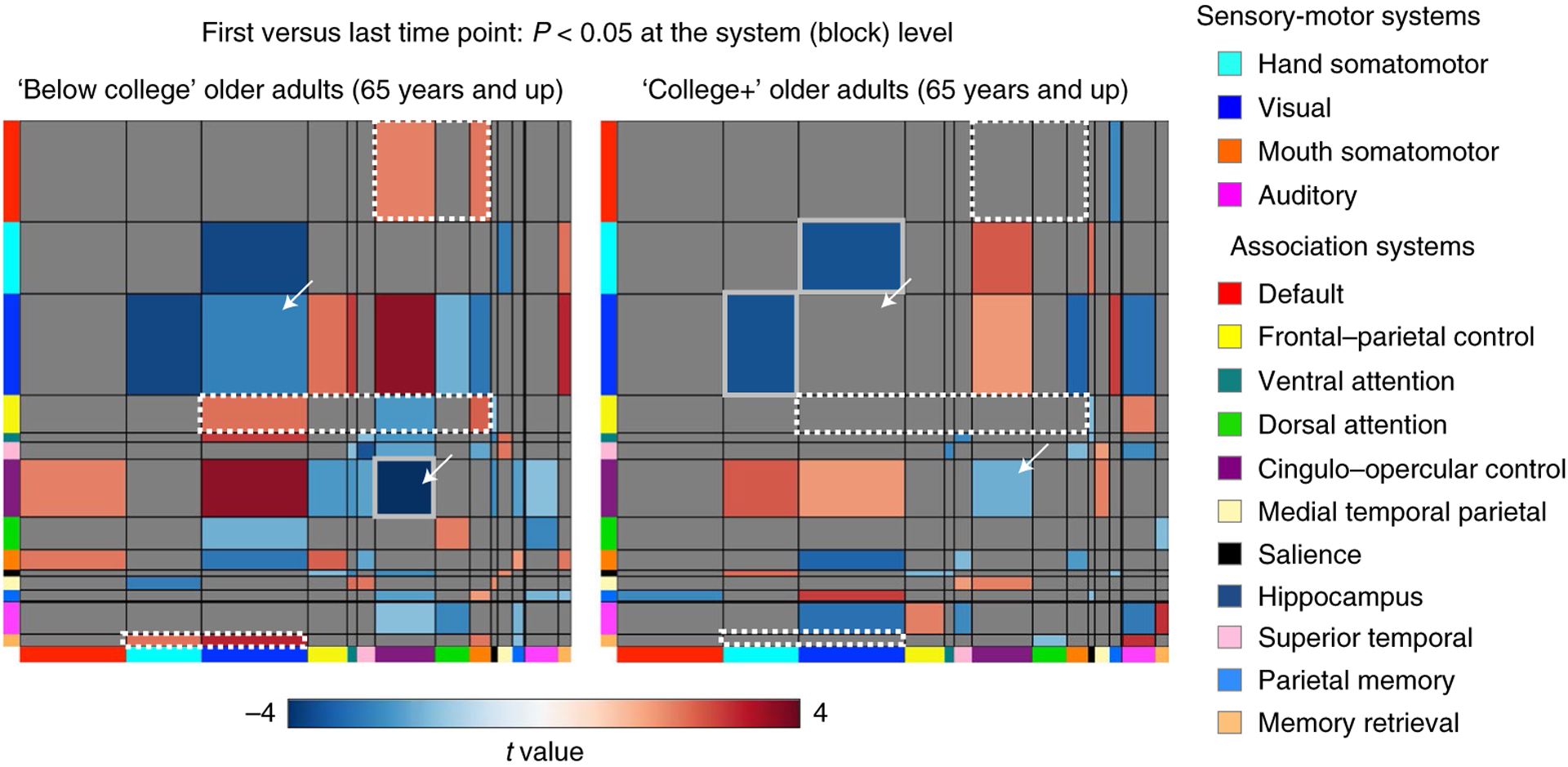
Network matrices comparing changes in resting-state correlations among older adults (65 years and over) with and without college degrees. Matrices depict significant changes in resting-state correlations (paired *t*-tests between baseline and the last time point) at the system level for older adults (65 years and over) in each education group. We highlight certain larger circuits that appear to exhibit education-related differences in patterns of change over time. This includes changes (typically increases) in between-system correlations involving regions of the default system, the frontal–parietal control system and the memory-retrieval system (white dotted borders) and decreases in within-system correlations of cingulo–opercular control and visual systems (white arrows), all of which are evident in ‘below college’ older adults but less so in ‘college+’ older adults. Some of these changes also contribute to changes in brain system segregation that were more evident in this ‘below college’ older adult group. Permutation was used to identify which system-level effects (blocks) were significant within each education group (see Methods for permutation details). Within each group, system-level changes (calculated by paired *t*-tests) that were not significant (cutoff of *P* < 0.05) (based on 10,000 permutations) are in gray, and the effects that survived false discovery-rate correction (at *P* < 0.05) are enclosed by solid gray borders.

Brain system segregation is a summary of the entire brain’s network organization. Further examination revealed system-specific distinctions in the observed network changes as a function of educational attainment. Fig. 4 illustrates that older adults in both education groups exhibit changes in resting-state correlation within and between partially overlapping brain systems over time. However, differences in RSFC changes between education groups were also observed, in particular, among brain systems that support integrative processing (that is, association systems^57^). These differences include greater changes in relationships involving the default system and frontal–parietal control system among ‘below college’ older adults, which were less evident in ‘college+’ older adults (white outlines in Fig. 4; also see section 1.3 in the Supplementary Information and Supplementary Fig. 2 for further analyses).

### Brain system segregation decline predicts impending cognitive and functional impairment

**Fig. 5:**
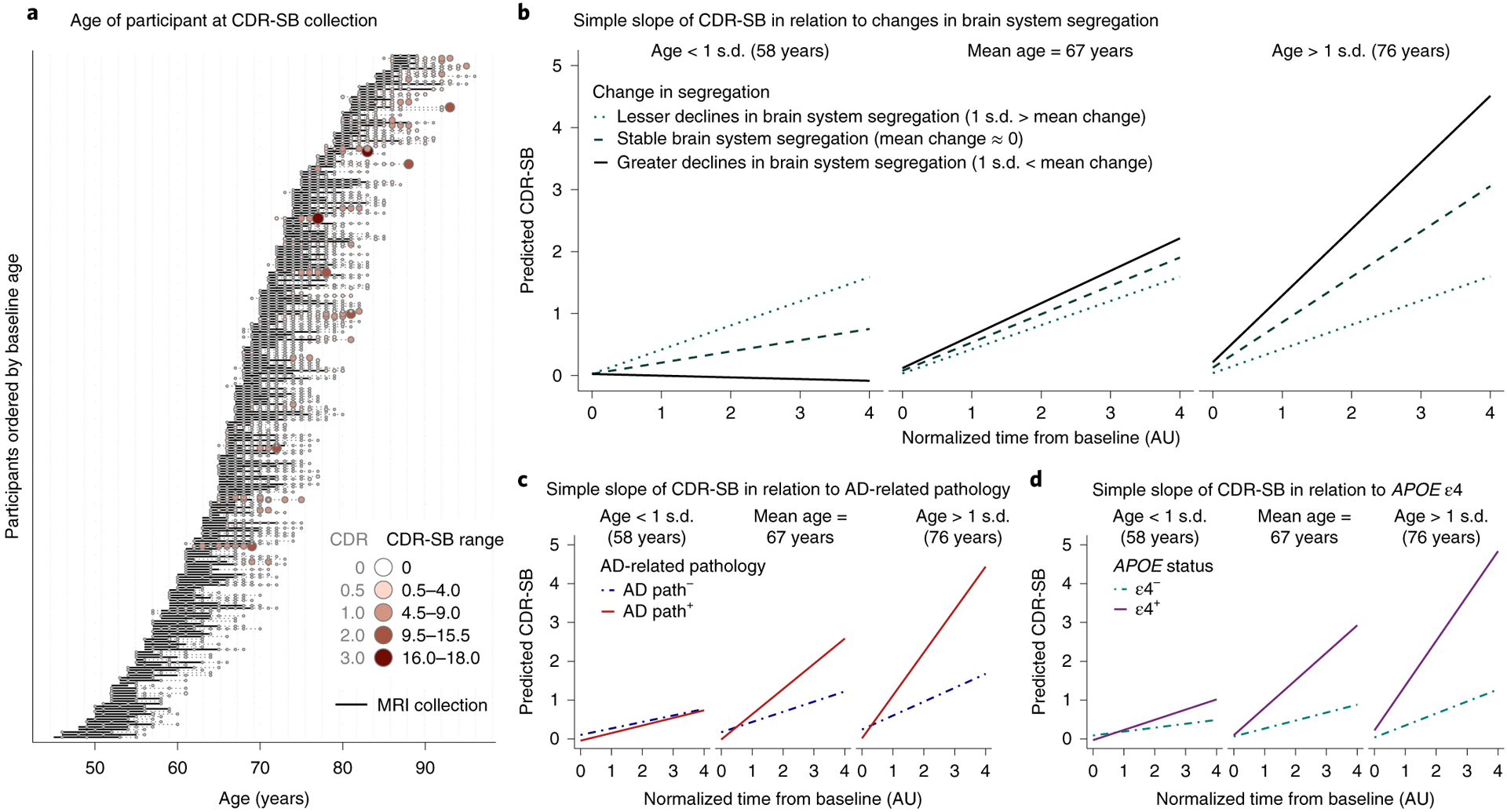
Changes in dementia severity are predicted by changes in brain system segregation, independently of AD-related brain pathology and *APOE* ε4 status and independently of education. **a**, Data from participants’ clinical sessions (CDR-SB data collection) that were collected within 1 year of baseline MRI scans and any time after (including sessions after their last MRI (resting-state) scans) were included. In total, 96% of participants had 3–17 available clinical sessions. The size and color of circles represent the magnitude of CDR-SB scores, binned according to global CDR scores (larger size and darker red represent higher CDR-SB scores; higher CDR-SB indicates greater cognitive or functional impairment). The corresponding global CDR ratings for the range of CDR-SB is also provided for ease of interpretation (section 1.4.1 in the Supplementary Information). For each participant, the dark black line represents the period of time between baseline and the last MRI scan. **b**–**d**, Simple slope plots depict the predicted slope of CDR-SB at three representative ages: 58, 67 and 76 years. **b**, Greater declines in brain system segregation (solid line) are associated with higher CDR-SB in older adults. Similar patterns, although statistically not significant in the present sample (see text), are observed for the presence of baseline AD-related pathology (path) (aggregate category based on the presence of elevated CSF pTau and/or elevated cortical amyloid levels; the solid line indicates the presence of pathology) (**c**) and positive *APOE* ε4 carrier status (the solid line indicates the presence of AD genetic risk) (**d**), separately. The depicted relationships (**b**–**d**) were not altered by structural changes (longitudinal measures of whole-brain gray matter cortical thickness or mean hippocampal volume). Further, these relationships were evident irrespective of participants’ educational attainment.

While studies have established relationships between brain system segregation and cognitive ability (for example, refs.^33,43,47^), the long-term prognosis of changes in brain network organization during adulthood is unknown. To determine whether the observed changes in brain network organization were predictive of impending clinical events, clinical data were examined, including data collected beyond the last available MRI (resting-state) scan (Fig. 5a depicts when clinical sessions occurred with respect to MRI sessions). The majority of participants had clinical data available beyond their last available MRI scan (*n* = 238; on average up to 3.77 years after the last scan, s.d. = 2.02 years, maximum = 10.10 years). Including these clinical sessions allowed us to examine whether brain network changes predicted future cognitive and functional changes. CDR sum of boxes scores (CDR-SB^58^), a clinical measure of cognitive and functional impairment (dementia severity) derived from six categories (Methods) were obtained for each individual, where higher CDR-SB reflect greater levels of impairment. CDR-SB was chosen to detect changes in impairment given its wider range of scores as compared to global CDR (0–18 versus 0–3), thus providing improved capability to differentiate levels of impairment. Supplemental analyses using participant’s global CDR scores in place of CDR-SB yielded qualitatively comparable results (section 1.4.1 in the Supplementary Information and Supplementary Fig. 3).

A linear mixed-effects model predicting CDR-SB revealed a significant three-way interaction between time, age at baseline and change in brain system segregation (observed difference between baseline and the last available measurement of brain system segregation; Methods) (*F*_1,258_ = 4.292, *P* = 0.039, CI_95%_ = (−0.033, −0.001)). This model and all subsequent reported models controlled for sex, average head motion, time (in days) between baseline and the last MRI scan and also education group. In older adults, greater declines in brain system segregation were associated with greater future cognitive impairment.

We next investigated whether changes in brain system segregation explained unique variance in the changes in cognitive impairment beyond known moderators of cognitive decline. Including measures of baseline AD-related pathology and *APOE* status in the model revealed that both AD-related pathology (*F*_1,233_ = 3.277, *P* = 0.072, CI_95%_ = (−0.041, 0.002)) and *APOE* status (*F*_1,234_ = 3.057, *P* = 0.082, CI_95%_ = (−0.040, 0.002)) marginally interacted with time and age in predicting changes in CDR-SB (Fig. 5c,d), while changes in brain system segregation significantly interacted with time and age in predicting changes in CDR-SB (*F*_1,242_ = 7.957, *P* = 0.005, CI_95%_ = (−0.052, −0.010); Fig. 5b). None of the higher-order interactions (four or five way) that included brain system segregation together with AD-related genetic risk and baseline pathology were statistically significant or marginally significant in predicting CDR-SB (*F*_s_ < 2.608, *P*_s_ > 0.108).

Together, the results suggest that changes in brain system segregation impact cognitive and functional status through a pathway independent of AD-related genetic risk or pathology. However, given the number of terms in the model that includes five independent variables (not including covariates), the present sample may be underpowered to detect higher-order interactions between these variables (for example, four-way or five-way interactions; Statistical analysis in the Methods). Based on the hypothesis that there may exist additive consequences across these variables that were not revealed in primary analyses, a supplemental analysis was conducted to depict possible relationships between these variables. These comparisons revealed some plausible additive effects that were not captured by the present statistical models (section 1.5 in the Supplementary Information and Supplementary Fig. 4).

In addition to amyloid and tau burden, another common biomarker for dementia is neurodegeneration^21^. Measures of neurodegeneration can be estimated using structural changes (for example, changes in gray matter cortical thickness or hippocampal volume) measured by in vivo structural imaging^59,60^. However, including either longitudinal changes in mean gray matter cortical thickness or hippocampal volume as a covariate did not qualitatively alter the interaction between time, age at baseline and change in brain system segregation in predicting CDR-SB (*F*_*s*_ > 7.969, *P*_s_ < 0.005). Further, when change in brain system segregation was replaced by either change in cortical thickness or hippocampal volume as the independent variable interacting with time and age at baseline, these models did not significantly predict CDR-SB (*F*_s_ < 0.785, *P*_s_ > 0.377).

Importantly, accounting for changes in CDR-SB observed during the period of time between an individual’s baseline MRI and last MRI scans did not qualitatively alter the relationship between changes in brain system segregation and impending cognitive decline among older adults (interaction between time, age at baseline and change in brain system segregation, *F*_1,250_ = 7.273, *P* = 0.007, CI_95%_ = (−0.037, −0.006)). This demonstrates that changes in brain system segregation predicted future cognitive impairment beyond an individual’s clinical trajectory from the same time period that the brain measures were collected (for additional analyses, see section 1.4.2 in the Supplementary Information).

Notably, none of the observed relationships between changes in brain system segregation and changes in CDR-SB in any of the models were moderated by educational attainment (for example, primary model, interaction between education group, time, age at baseline and change in brain system segregation in predicting CDR-SB, *F*_1,250_ = 0.317, *P* = 0.574, CI_95%_ = (−0.014, 0.025)). While declining brain system segregation was more prominent in older adults with less education, individuals who exhibited brain network decline were more likely to exhibit future cognitive and functional impairment, irrespective of their educational attainment.

## Discussion

Functional brain network decline is greater in older adults without a college education than that in their college-educated peers. In addition, decreasing resting-state brain system segregation is predictive of impending cognitive and functional impairment (dementia severity) among older adult individuals, independent of known AD-related genetic risk factors and measures of brain pathology or cortical thinning. However, educational attainment does not moderate the link between brain network changes and changes in clinical outcomes. While an individual’s education relates to their brain network changes during older age, if and when brain network changes occur, the impact on cognition is equally devastating irrespective of educational attainment.

### Declining brain system segregation foreshadows impending cognitive decline in older adults

The segregation of large-scale resting-state brain systems supports brain function throughout the lifespan^41^. Previous research has revealed that individual differences in measures of brain system segregation are related to differences in cognitive ability, such as episodic memory and processing speed, among normative adult samples^33,47,61^. It has been unclear whether changes in brain system segregation translate to any clinically meaningful changes in cognition or functional status as an individual grows older. The present study demonstrates that declining brain system segregation during older age is predictive of impending changes in dementia severity, independent of AD-related genetic risk and neuropathology or cortical thinning in older adults. Interestingly, this relationship was evident for impending decline beyond the available scan sessions, in some cases up to 10 years following an individual’s final MRI scan. Further analyses revealed that the predictive utility of changes in functional brain network organization on future clinical decline was also independent of the trajectory of clinical changes that coincided with scan sessions.

It is important to acknowledge that clinical observations measured from an individual’s CDR-SB scores are not necessarily specific to AD but can also capture impairment related to other forms of dementia^62^. However, we focused here on AD given the targeted study population (recruited under studies from the Knight ADRC) and the richness of the dataset, which has available measures of AD-related genetic risk and biomarkers of AD-related neuropathology. *APOE* ε4 is a genetic risk factor for AD that contributes to altered amyloid formation and clearance^63^ and was shown to be associated with cognitive decline in healthy middle-aged and older adults^64,65^ and during all stages of AD^66,67^. As expected, the presence of at least one ε4 allele was predictive of impending decline across older adults in the present sample. Critically, however, declining brain system segregation explained cognitive decline independently of *APOE* status. This observation is consistent with the current understanding of AD prognosis, in which genetics alone do not completely predict disease development in typical AD (that is, late-onset AD). Instead, a combination of environmental, psychosocial and lifestyle factors^18^ likely interact to alter brain structure (and, as evidenced here, functional brain network organization), which can then manifest as cognitive and behavioral impairment when brain degradation is substantial.

Multiple abnormal brain changes define AD and cognitive impairment, including Aβ deposition^20,25^, the presence of tau neurofibrillary tangles^19^ and neurodegeneration^68^. Validated proxies of these brain changes have been incorporated into an influential model of major biomarkers of AD, which summarizes the presence of pathology and neurodegeneration (A/T/N: abnormal levels of Aβ deposition (‘A’), abnormal levels of tau (‘T’) and neurodegeneration (‘N’)^21^). The A/T/N model has catalyzed efforts for in vivo disease staging and classification (for example, ref.^69^). The present results demonstrate that changes in functional brain network organization and its downstream consequence are, at least in part, independent of the cascade of known pathological and neurodegenerative burdens that form the basis of existing models of AD and should be incorporated into future models of AD. Future studies examining AD risk and dementia risk more broadly should aim at more targeted examination of the interactive effects and relationships between changes in functional brain network organization and changes in brain pathology and neurodegeneration, and their collective contributions to preclinical and clinical cognitive impairment^70^ (section 1.5 in the Supplementary Information).

### Brain system segregation as a measure of ‘reserve’ in aging

Past studies have shown that, when compared to those with lower education, highly educated individuals can maintain cognitive and functional abilities despite harboring greater amounts of pathologic burden (for example, refs.^13,71,72^). These observations helped motivate the ‘reserve’ theory of aging, which postulates the presence of an undetermined substrate that helps resist cognitive dysfunction despite the presence of neuropathology^73^. Reserve has typically been indexed by an individual’s educational attainment, which itself often serves as a proxy for SES and other environmental factors. But what aspects of brain structure or function actually allow an individual to seemingly resist the impacts of pathology? We previously hypothesized that an individual’s functional brain network organization, and brain system segregation more specifically, may be a brain measure of ‘reserve’ (refs.^41,48^), and the present observations support this hypothesis. Lower education was associated with greater declines in brain system segregation, independently of baseline AD-related pathology or longitudinal measures of cortical thickness. However, educational attainment did not moderate the relationship between changes in brain system segregation and cognitive decline: some individuals with higher education also exhibited declining brain system segregation, and, when they did, they were not immune to the deleterious cognitive impact of this pattern of brain network change. The preceding observations are also consistent with the idea that education may be a crude index of other variables that modify an individual’s brain structure and function. For example, educational attainment has been linked to cognitive and intellectual engagement during adulthood, which were shown to relate to age-related cognitive differences (for example, refs.^74,75^) and predict dementia risk^76^. However, given the lack of explicit measurement of cognitive engagement in the present participant sample, we were not able to directly determine whether and how aspects of engagement during adulthood related to changes in brain system segregation (see below for additional discussion on this and related issues).

In contrast to RSFC brain network organization, longitudinal structural changes (that is, mean gray matter cortical thinning) in older adults did not vary as a function of educational attainment (section 1.2 in the Supplementary Information). The absence of a relationship between education and longitudinal structural changes in adult individuals is consistent with a number of reports examining these relationships^11,77^. Accordingly, the present observations provide evidence that changes in functional network organization are more sensitive to an individual’s environment than changes in brain structure (at least globally defined) and motivate focus on resting-state network organization as a target for understanding environment-related and experience-related brain plasticity in studies of brain aging.

The mechanism by which changes in brain system segregation occur are uncertain^41^. One possibility is that focal degeneration of specific network nodes and their downstream outcomes (for example, refs.^78,79^) may alter functional networks. There is some evidence that SES-related variables and cumulative experiences linked to an individual’s environments may relate to structural differences in specific brain regions^80–82^. Potentially in line with this idea, closer examination of the changes in functional brain network topology highlighted specific brain circuits that may be particularly vulnerable to environment-related changes during older age (Fig. 4). For example, differences in RSFC changes between education groups were most prominently observed across association systems that support integrative processing. In particular, changes existed in ‘below college’ older adults that were not evident in ‘college+’ older adults, including decreases in RSFC in the cingulo–opercular system and greater between-system RSFC among nodes of the default mode, memory-retrieval and frontal–parietal systems (the latter aligning with a previous cross-sectional study^83^; see section 1.3 in the Supplementary Information for subsets of system changes in functional network organization). It remains to be determined whether and how specific structural changes may relate to specific functional network changes.

### Educational attainment is linked to social, economic and health disparities

Broadly, lower education is associated with greater incidence of dementia^6^ including AD^5^ but also greater incidence of mental health disorders^3,4^. Education does not dictate these brain health outcomes directly or in isolation; education has been shown to differentiate many important aspects of health, resources and lifestyle (for example, refs.^1,4,8,9,51^), a reality that was echoed in the present data (Fig. 1c–e and section 1.6 in the Supplementary Information). Because the complex relationship between education and economic opportunities broadly shapes an individual’s life course behaviors and environment, an important task is to understand what mediates the relationship between education and aging-accompanied brain changes^12^. Multiple candidate processes exist, many of which converge on biological pathways reflecting chronic exposure to environmental stressors, in which chronic stress results in elevated cumulative allostatic load that causes deterioration of the body and brain^84,85^. Admittedly, measures of physical and mental health included in the present study are neither clinically comprehensive nor complete. Further, absent in these measurements are deeper descriptions of lifestyle-level differences including nutrition and leisurely exercise (for example, refs.^86,87^), health behaviors (for example, nicotine and alcohol use^88^), cognitive and intellectual engagement (for example, refs.^74,75^) and access to or utilization of healthcare resources^89^, all of which likely play a critical role in stress and brain aging. Finally, an individual’s educational attainment can often be linked to environmental factors defining their childhood. Similar to the cumulative effects of childhood and adulthood adversity on health and mortality^2^, early life experiences from infancy to adolescence relate to brain structure and cognition in older age^81^. While the present observations do not differentiate early life from present experiences, an earlier study by members of our group reported that parental education (that is, childhood SES) did not attenuate the observed cross-sectional relationship between adult SES and brain system segregation^48^. Thus, it is likely that educational attainment and childhood experience have additive and unique effects on changes in brain network organization over the lifespan.

The present results fit with multiple lines of evidence demonstrating education-related disparities in brain health during advanced age (for example, refs.^5,6^) but offer a measurable feature of brain function, changes in which also signal the risk of impending cognitive decline before its occurrence. An important goal of follow-up work will be to understand the complex relationships that intertwine social determinants of health, longevity, lifestyle factors and changes in brain network organization^16^. Ultimately, a deeper understanding about the interplay between one’s environment and the brain could fill in the missing links between broader psychosocial societal factors and dementia risk, prevalence and prevention (for example, ref.^90^).

### Limitations

Using graph theory to analyze brain networks necessitates selection of multiple preprocessing and analytic parameters^29,30^. Supplemental analyses confirmed that the reported results were not limited to our decisions regarding the trade-off between resting-state data quality and quantity (section 1.7.1 in the Supplementary Information) or graph construction (that is, possible age-related differences in node definition; section 1.7.2 in the Supplementary Information and Supplementary Fig. 5). However, continued examination of longitudinal resting-state data will be crucial not only to confirm but also to better understand the properties of brain network organization that underlie the reported results.

The present dataset drew from a broader Knight ADRC dataset pool that is relatively diverse and representative of the catchment area where the data were collected. However, as is the problem with many neuroimaging studies, the final participant sample that passed rigorous data quality control included few participants with very low education (less than high school) and also included fewer non-white participants than would be expected based on the broader Knight ADRC data sample. In addition to possible selection biases due to health or education, longitudinal measurement also exposes the data to attrition bias, in which individuals with poorer health and, by association, poorer cognition, are less likely to remain in the study. Altogether, the detrimental effect of lower education on the declining trajectory of brain system segregation and the latter’s relation to clinical decline are likely underestimated given the under-representation of adults with very low education and health disparities that are prevalent among individuals with lower education.

### Conclusion

Older adults who never completed a college degree exhibit greater declines in resting-state brain system segregation, a measure of large-scale network organization and function. Declining brain system segregation predicts impending cognitive and functional impairment beyond known AD-related biomarkers of pathology and genetic risk and irrespective of an individual’s educational attainment. These observations demonstrate that changing functional network organization is an important preclinical warning signal of cognitive impairment that is not captured by measures of brain structure or pathology. Future studies should aim at both further elucidating the time course of brain network changes relative to clinical decline but also identifying environmental factors that mediate the relationship between an individual’s educational attainment and changes in their brain network organization. These developments would help establish causal pathways and identify modifiable targets for intervention. The urgency for continued work in this area cannot be overstated in the face of population aging, the prevalence of AD and other dementias that accompany increasing age, and growing health disparities among economically disadvantaged individuals.

## Methods

### Participants

Adult participant data were provided by the Knight ADRC at Washington University in St. Louis. Written informed consent was obtained from all participants in the study at the time of enrollment. Each of the datasets obtained from the Knight ADRC was collected under a study that had been approved by the Human Research Protections Office of Washington University in St. Louis. The data analysis included in the present work was approved by the Institutional Review Board of the University of Texas at Dallas. Participants’ data were only included in the present study if they had available (1) a minimum of two resting-state fMRI scan sessions to allow longitudinal functional brain network analysis and (2) demographic (age and self-reported sex) and education information; a total of 417 participants from the Knight ADRC satisfied the criteria. Participants’ structural and resting-state scans underwent structural and fMRI preprocessing, motion processing and surface mapping. Following initial processing, 266 participants had two or more sessions of data that passed all quality checks (QCs) listed in the Neuroimaging section below. Of the participants who were excluded from subsequent analysis, 28 participants failed preprocessing QCs (for example, poor skull stripping or FreeSurfer surface estimates due to artifacts in their T1 images), 116 participants failed the motion-processing QC (that is, they did not have adequate data after motion ‘scrubbing’) and seven participants failed the surface-mapping QC. Lastly, a single participant with a baseline CDR score^54^ of 1 was excluded because of their clinical diagnosis of mild dementia at baseline.

The final sample of 265 participants were 45–86 years old (*n* female = 154) at their baseline resting-state scan (mean age = 67.01 years, s.d. = 9.26 years). Variables associated with clinical status, AD-related pathology, cardiovascular health, neurological health and mental health were measured during separate data-collection sessions (for example, PET sessions). Data sessions closest to a participant’s baseline resting-state scan session were used for purposes of analysis (81% of data from these separate experimental sessions were collected within 1 year of the baseline resting-state scan session). The sample was largely cognitively normal, with 240 participants rated as cognitively normal at baseline on the CDR, corresponding to a CDR of 0; 25 participants had a CDR of 0.5, indicating the presence of very mild dementia. See Table 1 for a breakdown of other variables across education groups.

### Educational attainment and grouping

Across participants, the range of self-reported education time was between 6 and 22 years (education time above 22 years was recoded as 22 years), with four participants reporting less than 12 years of education (that is, they did not complete high school). While education time was collected as a numerical variable, each education year is not a uniform measure. The difference between 13 and 14 years of education is likely minimal, as both represent an individual completing some college. However, the difference between 15 and 16 years of education typically reflects the difference between someone with or without a college degree.

Importantly, the categorical distinction of having a college degree or not was shown to confer significant socioeconomic advantages, even when accounting for economic costs to obtain a degree^51^. This distinction is evident across large segments of adulthood, and this effect spans various fields of study, whereby even relatively less economically lucrative majors from a four-year degree translate into economic advantages^91^. Accordingly, for each participant, their self-reported time of formal education was converted into a variable coding for college degree attainment, for which 16 or more years of formal education was categorized as ‘college+’, as it approximates the time when most people complete a college degree. Those with fewer than 16 years of formal education were categorized as ‘below college’ (see section 1.8 in the Supplementary Information and Supplementary Figs. 6 and 7 for further analysis and discussion regarding categorization of education).

### Neuroimaging

Each participant had two or more resting-state scan sessions available, enabling longitudinal comparisons of their functional brain networks. On average, a participant’s second scan was 3.22 years (s.d. = 1.55 years) after their baseline scan, with their third, fourth and fifth scans (if available) being 5.70 years, 6.78 years and 8.05 years (s.d. = 0.43–1.62 years) after their baseline scan (see Fig. 1a and the embedded table for the spread of longitudinal data across participants and a breakdown of the number of scans in relation to the number of participants). For each MRI session, a T1-weighted structural image was also collected and used when preprocessing resting-state fMRI data for each scan session.

#### Structural imaging acquisition and preprocessing.

T1-weighted images (magnetization-prepared rapid-acquisition gradient echo sequence) were processed using FreeSurfer 5.3 to obtain measures of the participant’s cortical surface and cortical thickness at each time point. Manual examination was performed on all FreeSurfer outputs, and, when necessary, manual editing (that is, control points, white and pial surface edits) was performed to ensure accurate construction of the cortical surface. Initial segmentation and manual editing were available with each of the structural images obtained from the Knight ADRC; every T1 segmentation (gray matter, white matter and CSF tissue mask) and surface generation (pial and white surfaces) of every participant were rechecked, and additional manual editing was performed based on procedures developed by our laboratory^92^.

#### Resting-state fMRI acquisition and preprocessing.

RSFCs were computed using functional brain images that are sensitive to blood-oxygen-level-dependent (BOLD) activity obtained using an echo-planar sequence (TR = 2,200 ms, TE = 27 ms; flip angle = 90°; FOV = 256 × 256 mm; 36 slices, interleaved acquisition; resolution = 4 × 4 × 4 mm). During each of the resting-state functional scans, participants were instructed to fixate on a visual cross-hair, remain still, keep their eyes open and not fall asleep. In general, each imaging session included two runs of resting-state scans, each consisting of 164 volumes. However, there were five sessions of data that included extra resting-state scans (four with three runs, and a single session had four runs); participant data for each of these sessions were inspected and processed along with the first two runs available for that session.

BOLD images (resting state) corresponding to the same session as each of the structural images described above were processed using a standard fMRI preprocessing pipeline using Nipype 0.8.0, including the following steps: (1) slice-timing correction to remove odd–even slice intensity differences due to interleaved acquisition, (2) rigid body correction for estimating and correcting head movement between volumes and (3) realignment to the T1-weighted image from the same session. All steps were performed using FSL 5.0.2.2, except for realignment between volumes and rigid body correction, for which SPM8 was used, as it provided more accurate estimates in our sample. The intensity of BOLD data was scaled to a mode of 1,000 (ref.^93^).

Following standard fMRI preprocessing, additional RSFC-specific processing and head-motion correction were applied to reduce spurious variance from non-neuronal activity, including the following steps: (1) demeaning and detrending; (2) multiple regression to remove variance related to whole-brain signal, ventricular signal, white matter signal, their derivatives and the ‘Friston 24’ motion regressors; (3) removing and interpolating motion-contaminated frames that were flagged as having FD > 0.3 mm or data between two contaminated frames that were less than five frames apart (‘scrubbing’ (ref.^94^)); (4) bandpass filtering (0.009–0.08 Hz); (5) removing the interpolated frames that were used to preserve the time series during regression and bandpass filtering.

While a part of the global signal may contain variance related to general levels of arousal and genuine neural activity (for example, refs.^95,96^), there is considerable evidence that a major component of the global signal includes spatially nonspecific signal artifacts related to head motions^94,97–99^. Failure to explicitly remove the global signal prohibits the control of these known influences of artifact^94,98,100^. As no method presently exists for denoising known artifactual signals while retaining ‘real’ signals^101,102^, the alternate option of retaining the global signal in each participant is likely to result in misestimation of correlations and the resultant network estimates. Accordingly, we employed data-censoring (‘scrubbing’) and signal-processing procedures, which, together with global signal regression, were shown to best reduce global and distance-dependent artifacts^98,103^.

RSFC processed data were registered to the fs_LR (32k) surface-based atlas for analysis^104^. Using the transformation matrix and deformation maps generated during preprocessing of the corresponding structural data, volumetric functional data were resampled to the fs_LR surfaces through a one-step transformation. Lastly, surface-mapped functional data on fs_LR surfaces were smoothed using a Gaussian smoothing kernel (*σ* = 2.55).

To ensure that varying amounts of data across individuals and sessions did not alter the estimation of their brain network, the number of frames in each session was fixed at 100 frames. Note that the results in the present study remained largely the same when using more stringent frame thresholds (that is, discarding participants that did not have at least 125 and 150 frames; section 1.7 in the Supplementary Information). However, using a higher frame threshold reduced the number of participants retained, reducing the power to test secondary models; thus, the 100-frame threshold was used to report the primary results.

### Brain network construction

For each session of surface-mapped resting-state data, a functional correlation matrix was generated with 349 surface-based nodes that were defined from previous boundary-based analyses^33,105^ and were labeled based on their spatial overlap with the vertex-wise community map published by Power et al. (not the 264 nodes^39^). This is the same approach that we adopted in previous publications^33^ with an additional constraint (see step 3 below; as in refs.^44,48^). Briefly, nodes were constructed using the following steps: (1) identifying putative area centers that were at least 8 mm apart based on an RSFC-boundary map^105^, (2) creating disks with a radius of 3 mm around the identified area centers to avoid area borders that may exhibit more variance between individuals, and (3) discarding nodes that were in areas of low signal intensity in the original data that were used to create the boundary map (<800 (ref.^44^)). All vertices within a node disk were identified based on their spatial overlap with an a priori vertex-wise community map in the same fs_LR space^39^, where each disk was labeled with a functional system based on a winner-take-all approach.

The BOLD time series of all vertices within each node were averaged to obtain the node’s mean time series. A correlation matrix (brain network) was constructed by computing the pairwise Fisher’s *z*-transformed Pearson’s correlation of each of the 349 nodes.

As described above, while the use of global signal regression in RSFC processing has attracted varying opinions, it is objectively effective for minimizing motion-related artifacts^94,99^ and is the recommended method to reliably remove global respiration-related artifacts when direct estimates of respiration are unavailable^101^. However, due to the application of global signal regression during preprocessing, negative correlations may be artifactually introduced through this procedure^106,107^; therefore, similar to past studies^39,48^, negative correlations were excluded in the final brain network matrices (negative values were set to zero). Computing brain system segregation (described below) does not require a sparse network matrix, which avoids ambiguous parameter selection such as edge density thresholding. Therefore, weights of matrices were unaltered, and additional edge density thresholding was not applied.

### Measures

#### Measurement of functional brain network organization.

##### Brain system segregation

System segregation measures the degree to which the functional brain network is organized into distinct subnetworks^33,41^. When referring to the measure here, we use the phrase ‘brain system segregation’ to insulate it from any sociopolitical connotation of segregation. Brain system segregation is calculated by taking the difference between mean within-system and mean between-system correlations as a proportion of mean within-system correlation, as noted in the following formula:

$$\text{Brain}\;\text{system}\;\text{segregation}=\frac{\frac{\sum_{w}^{W}Z_{\text{w}}}{W}-\frac{\sum_{b}^{B}Z_{\text{b}}}{B}}{\frac{\sum_{w}^{W}Z_{\text{w}}}{W}},$$

where *Z* is a Fisher’s *z*-transformed correlation value, representing an edge between a pair of nodes. *Z*_w_ represents the edges (correlations) between pairwise nodes that belong to the same system (within system), *Z*_b_ represents the edges between pairwise nodes that belong to different systems (between system), *W* is the total number of within-system edges across all subnetworks, and *B* is the total number of between-system edges across all subnetworks. In the past, we expressed this calculation as shown in the following equation (as in ref.^33^):

$$\text{Brain}\;\text{system}\;\text{segregation}=\frac{{\bar{Z}}_{\text{w}}-{\bar{Z}}_{\text{b}}}{{\bar{Z}}_{\text{w}}},$$

where ${\bar{Z}}_{\text{w}}$ is the mean within-system correlations, and ${\bar{Z}}_{\text{b}}$ is the mean between-system correlations. The updated formula more accurately specifies the exact computation of the measure to minimize ambiguity. Higher brain system-segregation values correspond to brain networks with greater separation of constituent functional systems.

#### Measures of brain structure.

##### Mean gray matter cortical thickness

A measure of brain structure was available at every time point when resting-state data were collected. Using the edited FreeSurfer segmentations, gray matter cortical thickness was estimated as the distance (in mm) between pial and white matter surfaces across the vertices on the cortical surface. The mean cortical thickness was calculated by averaging the cortical thickness measurement of the left and right hemispheres. Intracranial volume (ICV) was obtained from FreeSurfer to adjust the cortical thickness. Using non-ICV-adjusted cortical thickness yielded qualitatively similar results.

##### Hippocampal volume

At every time point when resting-state data were collected, left and right hippocampal volume measures (mm^3^) were extracted from FreeSurfer segmentation estimates and averaged together to form a mean hippocampal volume measurement. Participants’ hippocampal volumes were adjusted by their individual head sizes using their ICV obtained from FreeSurfer.

#### Measures of Alzheimer’s disease clinical status, pathology and genetic risk.

##### Clinical status

Clinical status was measured by the CDR scale^54^, which is based on scoring for six categories: memory, orientation, judgment or problem solving, community affairs, home and hobbies, and personal care. These categories represent both cognitive and functional behaviors. CDR was used to provide a global classification of dementia stage at baseline, and 25 participants (9%) were classified with a CDR of 0.5 at baseline, indicating the presence of very mild dementia. In the analysis predicting longitudinal brain system-segregation changes, the raw baseline CDR score (ranging from 0 to 0.5) was included as a covariate in the models predicting education-related longitudinal brain network changes.

Longitudinal CDR-SB was used as a dependent variable in the models examining longitudinal changes in cognitive impairment. CDR-SB was used as the primary dependent variable due to its finer resolution for differentiating mild cases of cognitive impairment (range, 0–18), which facilitates detection of change in clinically relevant cognitive decline^58^. See Supplementary Fig. 8 in section 1.9 of the Supplementary Information for distribution of longitudinal CDR-SB scores across the two education groups.

##### Alzheimer’s disease-related pathology

The presence of elevated cortical amyloid uptake and CSF pTau 181 levels was used to categorize participant’s AD-related neuropathology. Processed amyloid and CSF pTau values were provided by the Knight ADRC^108,109^. Mean cortical amyloid levels were measured with PiB, a PET imaging tracer that binds to fibrillar deposits of Aβ. A cutoff value of 1.42 SUVR using the cerebellar cortex as the reference region^108^ was used to identify participants with elevated levels of amyloid deposits (amyloid PiB^+^, *n* = 47; PiB^−^, *n* = 160). CSF pTau values were obtained by analyzing CSF samples using enzyme-linked immunosorbent assays (Innotest, Innogenetics^110^). Participants with a value above 67 pg/ml were categorized as having elevated levels of pTau (pTau^+^, *n* = 61; pTau^−^, *n* = 176)^109^. A large portion of the final sample had both of these variables available (*n* = 193 of 265 in the final sample); however, a subset was missing some form of AD-related pathology data (see Table 1 for missing data). Participants with one or more elevated AD-related pathology markers were categorized as positive for AD-related pathology.

##### *APOE* ε4 status

*APOE* genotyping was performed following standard procedures for extracting DNA from peripheral blood samples (see detail for *APOE* genotyping in ref.^111^). Participants with at least one copy of the ε4 allele were categorized as *APOE* ε4^+^ (*n* = 94, 36%).

#### Measures of cardiovascular health and mental health.

Each participant’s cardiovascular health was incorporated into analyses by including independent measures related to cardiovascular risk and cardiovascular incidents. Cardiovascular health was quantified as the proportion of cardiovascular-related variables that were available and met the following criteria: (1) BMI > 30, (2) recent or remote hypertension, (3) recent or remote hypercholesterolemia, (4) recent or remote incident of heart attack or cardiac arrest, atrial fibrillation, angioplasty or endarterectomy or stent, cardiac bypass procedure, pacemaker or congestive heart failure. All incident measures represented a binary distinction as to whether a participant had either recently or remotely experienced the particular health issue or had never experienced it (that is, absent) at the time of their first scan.

Measures of participant’s neurologic and mental health included details of traumatic brain-injury incidents and depressive symptoms, respectively. Traumatic brain-injury incidents were categorized and included in analyses as a categorical variable. Participants with any recent or remote incident of the following were categorized as positive for incidence of traumatic brain injury: traumatic brain injury accompanied by (1) brief loss of consciousness (<5 min), (2) extended loss of consciousness (≥5 min) or (3) chronic deficit or dysfunction. Ten participants were missing traumatic brain-injury-incident data. Depressive symptoms were measured by the GDS and included as a continuous variable in the analyses^112^. The scale has a possible range from 0 to 15 (0–4, no depression; 5–8, mild depression; 9–11, moderate depression; 12–15, severe depression). The present sample had a GDS score ranging from 0 to 8, with nine participants scoring between 5 and 8. One participant was missing GDS data.

#### Measurement of alternate socioeconomic variables.

##### Occupational socioeconomic index

Each participant’s self-reported occupation was matched to a corresponding occupation code in the US census and then assigned a sex-specific SEI based on predicted occupation prestige, a composite score reflecting one’s occupational wages, occupational education and wage–occupation–prestige index^52^. If an occupation listed had no direct match in the US census occupation code, the occupation code for the most related job was used. Two independent coders (C.A.C. and R.M.R.) examined occupations without direct occupation codes to reach a consensus coding. Any participants listing their occupation as homemaker or student were not coded (coded as N/A) because a prediction of occupation-related prestige is not available. As such, SEI values were available for 228 of 265 participants.

##### Neighborhood median household income (2011–2015 American Community Survey)

Each participant’s neighborhood income was estimated based on the median household income of the zip codes in which they resided. Beginning in 2010, the median household income of a zip code became available from the American Community Survey (ACS). Because some participants’ scans were collected before 2010, it was not possible to have individualized estimates based on the years in which the scan was collected for all participants. Instead, 5-year ACS data from 2011 to 2015 were used because they encompass the median scan date across all available scanning sessions (median scan date, 2011). Five-year ACS estimates were chosen over 3-year or 1-year ACS estimates because they covered a greater proportion of zip codes (that is, more areas are missing from 3-year or 1-year data) and used larger sample sizes to determine estimates. Not every zip code had 5-year ACS estimates available; therefore, some participants with zip code data did not have a matching neighborhood median household income estimate; thus they were excluded from the comparison in Fig. 1d. Neighborhood median household income data were estimated for 171 of 265 participants.

##### National area deprivation index (2011–2015 American Community Survey)

The national ADI represents the percentile ranking of neighborhood SES disadvantages, calculated using multiple variables (for example, home value, gross rent, percent of families below poverty level, percent of households without a motor vehicle^53^). ADI data used in the present study were obtained from https://www.neighborhoodatlas.medicine.wisc.edu^113,114^, calculated using 5-year ACS data from 2011 to 2015. ADI scores were calculated on the block group level, for which data were first linked to nine-digit zip codes. Nine-digit zip codes are sub-areas of five-digit zip codes. To estimate the ADI for a participant from the Knight ADRC, ADI scores of all nine-digit zip codes within the participant’s five-digit zip code were averaged together. ADI was available for 236 of 265 participants. Because ADI data are aggregated from nine-digit zip codes and multiple measures of ACS data, more participant ADI data were available than median household income data.

### Statistical analysis

The present study used a longitudinal design, in which the measure of time (that is, days from baseline) was included as a within-participant variable. Both analyses examining brain changes and cognitive impairment changes used a linear mixed-effects approach. First, in the analysis predicting longitudinal brain changes, linear mixed-effects models were used to examine how the dependent variable (for example, primary analysis, brain system segregation; supplementary analyses, cortical thickness) was predicted by time (normalized time from baseline; within participant) and its interaction with education group (between participant) and age at baseline (between participant). Age at each scan was not used as a measure of time to allow us to investigate the interaction between age and time. For the time variable, the number of days that a scan was collected after that individual’s baseline scan was normalized and adjusted to the point where 0 is the baseline.

In the primary model, in-scanner head motion (mean FD; within-participant variable collected at each longitudinal time point) and sex (between-participant variable) were included as covariates. The linear mixed-effects model was calculated as follows:

$$Y_{ij}=\gamma_{00}+\gamma_{01}\left({\text{sex}}_{j}\right)+\gamma_{02}\left({\text{age}}_{j}\right)+\gamma_{03}\left(\text{edu}\;{\text{grp}}_{j}\right)+\gamma_{04}\left({\text{age}}_{j}\times \text{edu}\;{\text{grp}}_{j}\right)\;+\gamma_{10}\left({\text{time}}_{ij}\right)+\gamma_{20}\left({\text{motion}}_{ij}\right)+\gamma_{11}\left({\text{time}}_{ij}\times {\text{sex}}_{j}\right)+\gamma_{12}\left({\text{time}}_{ij}\times {\text{age}}_{j}\right)\;+\gamma_{13}\left({\text{time}}_{ij}\times \text{edu}\;{\text{grp}}_{j}\right)+\gamma_{14}\left({\text{time}}_{ij}\times {\text{age}}_{j}\times \text{edu}\;{\text{grp}}_{j}\right)+\mu_{0j}+\mu_{ij}\left({\text{time}}_{ij}\right)+\epsilon_{ij}$$

where *Y*_*ij*_ denotes brain system segregation for each participant *j* at time *i*, *γ* denotes the estimated fixed-effect coefficients, *μ* denotes the estimated random-effect coefficients, *ϵ* denotes the residual for each participant *j* at time point *i*, and edu grp is the education group. Sex and its interaction with time was included to account for the fixed effect of sex on the random effect of time. Following a similar approach, subsequent models included between-participant covariates (for example, AD-related pathology, cardiovascular health factor) and their interaction with time by entering additional terms *γ*_0*k*_ (covariate_*ij*_) and *γ*_1*k*_ (time_*ij*_ × covariate_*j*_) where *k* is the *k*th covariate. When a covariate was collected longitudinally (for example, motion, cortical thickness), it was included simply as *γ*_*k*0_

Multiple-comparison correction was applied when examining the two unique longitudinal brain change measures (brain system segregation and cortical thickness). After correction, the comparison would require a *P* value of 0.025 to be considered significant. The interaction between age, time and education predicting brain system segregation surpassed this corrected *P* value, while the three-way interaction predicting cortical thickness remained insignificant. All reported *P* values in the text are raw *P* values.

In the analysis on longitudinal changes in cognitive impairment, similar linear mixed-effects models were constructed, with the dependent variable being CDR-SB or CDR (section 1.4.1 in the Supplementary Information). Changes in brain system segregation were defined as the observed difference between brain system segregation measured from the last time point and baseline, as opposed to estimated changes from mixed models. Although a substantial number of participants had more than two time points of resting-state data available, a notable portion only had two time points; therefore, estimates for these individuals were more prone to shrinkage (that is, coefficients shifted more toward population values than within-participant least square estimates^115^). Accordingly, difference scores were used to ensure a consistent means to obtain within-person changes across all participants, which also meant that the conclusion from the model predicting CDR-SB was based on actual changes in brain system segregation instead of estimated changes from a separate mixed-effect model. Furthermore, when predicting changes in CDR-SB, in addition to sex and average head motion (baseline and last scan), time (in days) between baseline and the last MRI scan was included to account for the varying length of time available to quantify changes in brain system segregation. Education group was also included as a covariate in all models except when its interaction was tested.

Normality of the dependent and independent variables was examined qualitatively using Q–Q plots, in which normality was relatively high for most variables. The normality of brain system segregation increased through quadratic transformation although not substantially. We repeated the analyses using squared brain system segregation as the dependent variable, and the results presented in Table 2 remained qualitatively the same. CDR-SB is not normally distributed due to the higher number of participants with 0 or relatively low CDR-SB scores. Following a previous study examining CDR-SB in relation to continuous time scale^25^, when quadratic time was included in the model predicting CDR-SB, the interaction between time, age at baseline and brain system-segregation change remained qualitatively the same.

Residuals of the linear mixed-effects models were examined to ensure that they were not correlated with the fitted values of the fixed-effects portion of the model, nor were the residuals associated with the independent variables (for example, age at baseline, education group).

### Block-level matrix comparisons across time

Longitudinal RSFC changes in older participants (65 years and over) within each education group were conducted by comparing their RSFC matrix from the first time point to that at the latest time point. Each node-by-node RSFC matrix at each time point was combined into ‘blocks’ based on predefined system labels^33,39^. Correlations among nodes from the same system were averaged together to form within-system blocks, and correlations among nodes across every pair of systems were averaged together to form between-system blocks. Observed block-wise comparisons across time were computed using paired *t*-tests (last scan versus first scan). Permutation was used to generate null distributions of block-level *t* statistics (permutation *n* = 10,000). Each permutation iteration shuffled the matrices within an education group across participants and time points, and the *t* value from a paired *t*-test using the permuted sample was recorded. For each block, the *P* value was calculated as the proportion of sampled statistics more extreme than the actual statistic. A two-tailed *P* value less than 0.05 was considered significant, and *t* values for each statistically significant block were visualized (blocks that survived false discovery-rate correction were further highlighted).

### Software

Linear mixed models and data visualization were carried out in R 3.6.0 using the following packages: lmer4 (1.1–26) for linear mixed-effects models; emmeans (1.3.5) for extracting model estimates; ggplot2 (3.3.3), tigris (0.9.4) and choroplethrZip (1.5.0) for visualization. Block-level matrix permutation was conducted in MATLAB 2019a using in-house scripts. Longitudinal spring-embedded graphs were generated using SoNIA (1.2.2) and exported using Cytoscape (3.2.0) to generate high-resolution images. Visualization of nodes on cortical surfaces were generated using Connectome Workbench (1.3.2).

### Reporting Summary

Further information on research design is available in the Nature Research Reporting Summary linked to this article.

## Supplementary Material

Supplementary Information

## Figures and Tables

**Table 1:** Demographic, health and AD-related information

Variables	Below college (*n* = 92)	College+ (*n* = 173)	Total (*n* = 265)	*P* value
**Demographic**				
Age, mean, years (s.d.)	68.07 (8.41)	66.45 (9.66)	67.01 (9.26)	0.176
Sex, *n* female (%)	65 (70.7%)	89 (51.4%)	154 (58.1%)	0.004
Race, *n* white (%)	82 (89.1%)	157 (90.8%)	239 (90.2%)	0.837
Education, mean, years (s.d.)	12.77 (1.39)	17.43 (1.43)	15.82 (2.63)	<0.001
**Cardiovascular health**, **neurological health and mental health**				
BMI^a^, mean, kg/m^2^ (s.d.)	27.47 (4.55)	26.51 (4.48)	26.85 (4.52)	0.102
Recent or remote hypertension^a^, *n* (%)	36 (39.1%)	73 (42.2%)	109 (41.1%)	0.725
Recent or remote hypercholesterolemia^a^, *n* (%)	44 (47.8%)	65 (37.8%)	109 (41.3%)	0.148
Recent or remote cardiovascular incidents^a^, *n* (%)	12 (13.0%)	17 (9.9%)	29 (11.0%)	0.576
Recent or remote traumatic brain injury^a^, *n* (%)	6 (6.7%)	13 (7.8%)	19 (7.5%)	0.948
GDS^a^, mean score (s.d.)	1.36 (1.56)	0.91 (1.28)	1.06 (1.40)	0.012
**Baseline clinical status, AD-related genetic risk and pathology**				
CDR-SB^a^, mean score (s.d.)	0.19 (0.53)	0.19 (0.64)	0.19 (0.60)	0.976
CDR^a^ > 0, *n* (%)	11 (12.0%)	14 (8.1%)	25 (9.4%)	0.422
*APOE* ε4^+^, *n* (%)	32 (35.2%)	62 (36.7%)	94 (36.2%)	0.914
CSF pTau^a^, mean, pg/ml (s.d.)	55.95 (34.23)	60.12 (31.94)	58.68 (32.74)	0.352
CSF pTau^+a^, >67 pg/ml, *n* (%)	15 (18.3%)	46 (29.7%)	62 (26.1%)	0.080
PiB amyloid^a^, mean cortical SUVR (s.d.)	1.30 (0.58)	1.32 (0.64)	1.31 (0.62)	0.772
PiB amyloid^+a^, >1.42 SUVR, *n* (%)	17 (23.3%)	30 (22.4%)	47 (22.7%)	1.00
**Other**				
In-scanner motion^b^, mean FD (s.d.)	0.21 (0.07)	0.21 (0.08)	0.21 (0.07)	0.478

The mean (s.d.) or counts (%) of numerical or categorical variables are shown for each education group and the entire sample. Statistical differences between the two education groups were calculated for continuous and categorical variables using *t*-tests and *χ*^2^ tests, respectively. Missing data for ‘below college’ (BMI, *n* = 1; traumatic brain injury, *n* = 3; GDS, *n* = 1; CSF pTau, *n* = 10; PiB amyloid, *n* = 19; *APOE*, *n* = 1). Missing data for ‘college+’ (BMI, *n* = 1; hypercholesterolemia, *n* = 1; cardiovascular incidents, *n* = 2; traumatic brain injury, *n* = 7; *APOE*, *n* = 4; CSF pTau, *n* = 18; PiB amyloid, *n* = 39). Abbreviations, SUVR, standard uptake value ratio; FD, frame displacement.

a Variables that were measured at different times than the functional MRI (fMRI) scans. The closest point of data collection of each measure to that of the baseline fMRI scan was used (see text for details).

b In-scanner motion from all available scanning sessions, compared across the two education groups.

**Table 2:** Linear mixed-model interaction statistics between time, education group and age at baseline on brain system segregation, accounting for different covariates

Covariates	Interaction between time, education group and age at baseline				
	*n*	*F*	*P*	2.5% CI	97.5% CI
**Accounting for participant demographics**					
Head motion^a^ and sex	265	6.814	0.010	−0.021	−0.003
Head motion^a^, sex and race^b^	265	6.399	0.012	−0.021	−0.002
**Accounting for baseline measures of clinical status, AD-related genetic risk and pathology, cardiovascular health, neurological health, and mental health**					
Clinical status, head motion^a^ and sex	265	6.652	0.011	−0.021	−0.003
*APOE* status, head motion^a^ and sex	260	6.393	0.012	−0.021	−0.003
AD-related pathology, head motion^a^ and sex	251	3.949	0.048	−0.020	−0.000
Cardiovascular health, head motion^a^ and sex	265	6.836	0.010	−0.021	−0.003
GDS, head motion^a^ and sex	264	7.092	0.008	−0.022	−0.003
TBI, head motion^a^ and sex	255	6.559	0.011	−0.021	−0.003
Clinical status, *APOE* status, AD-related pathology, cardiovascular health, GDS, TBI, head motion^a^, sex and race^b^	240	3.257	0.073	−0.020	0.001
**Accounting for longitudinal measures of brain structure**					
Cortical thickness^a^, head motion^a^ and sex	265	5.915	0.016	−0.021	−0.002

a Variables are longitudinal measures collected at the same time as fMRI scans (for example, head motion, cortical thickness).

b Race is categorized as white and non-white; <10% of the present sample is non-white. CI, confidence interval.

## Data Availability

Data include patient information and are private and unsuitable for public deposition. Imaging and behavioral data are available to investigators upon request and approval from the Knight ADRC Leadership Committee. The ADRC Leadership Committee meets on the second Monday of January, March, May, July, September and November each year. Data requests are approved on a rolling basis. Requests involving neuroimaging data should be submitted to the ADRC for preliminary review before the leadership committee meeting (director of the imaging core, T. Benzinger, benzingert@wustl.edu). Detailed instructions for making a request can be found at https://knightadrc.wustl.edu/Research/ResourceRequest.htm.
