# Supplementary material for: Long-term prognosis and educational determinants of brain network decline in older adult individuals
Source: Nat Aging. Author manuscript; Available in PMC 2022 Apr 4. (PMC8979545; doi:10.1038/s43587-021-00125-4)
Supplement: Supplementary Information [file NIHMS1787577-supplement-Supplementary_Information.pdf]

nature portfolio

https://doi.org/10.1038/s43587-021-00125-4

Supplementary information

Long-term prognosis and educational determinants of brain network decline in older adult individuals

In the format provided by the
authors and unedited

Supplementary Information (SI) for:

Long-term prognosis and educational determinants of brain network decline in older adult individuals

Micaela Y. Chan[1], Liang Han[1], Claudia A. Carreno[1], Ziwei Zhang[1], Rebekah M. Rodriguez[1], Megan LaRose[2], Jason Hassenstab[2], Gagan S. Wig[1,3]

[1]Center for Vital Longevity, School of Behavioral & Brain Sciences, University of Texas at Dallas, Dallas, TX, 75235, USA
[2]Department of Neurology, Washington University School of Medicine, St. Louis, MO, 63110, USA
[3]Department of Psychiatry, University of Texas Southwestern Medical Center, Dallas, TX, 75390, USA

Corresponding author: Gagan S. Wig, Ph.D.
Email: gwig@utdallas.edu

This PDF file includes:

SI Results (Figures S1 to S8)
SI References

1. Supplemental Results ________ ***2***

1.1 – Education and age moderate change in brain system segregation in cognitively normal adults ________ **2**

1.2 – Educational attainment does not moderate aging-accompanied changes in brain structure ________ **3**

1.3 – The relationship between education and changes in brain system segregation varies across different types of functional systems ________ **4**

1.4 – Brain system segregation decline predicts impending cognitive and functional impairment ________ **7**

1.4.1 – Changes in global CDR are predicted by changes in brain system segregation ________ 7

1.4.2 – Further examination of the temporal relationships between brain network and clinical changes ________ 8

1.5 – Changes in brain system segregation may exhibit additive effects with both APOE status and baseline AD-related pathology to predict long-term changes in CDR-SB ________ **9**

1.6 – The relationship between educational attainment and changes in brain system segregation persists while controlling for additional environment-related variables ________ **11**

1.7 – Examination of relationships across data parameters (amount of resting-state data per participant) and graph constructions (age-specific brain area parcellations) ________ **12**

1.7.1 – Impact of varying amount of resting-state data per participant ________ 12

1.7.2 – Age-group specific brain area parcellations to generate brain network nodes ________ 13

1.8 – Education attainment distinctions are well captured by the Below College vs. College+ categorization ________ **16**

1.9 – Distribution of CDR-SB over time across both education groups ________ **18**

2. Supplemental References ________ ***19***

1. SUPPLEMENTAL RESULTS

1.1 – Education and age moderate change in brain system segregation in cognitively normal adults

At the time of their baseline scans, the majority of participants were classified as cognitively normal based on the Clinical Dementia Rating (CDR): 240 participants with CDR=0, and 25 with CDR=0.5, with CDR at 0.5 indicating very mild dementia. Reanalyzing the mixed-effects model after excluding all participants who were assigned a CDR>0 at baseline did not qualitatively change the three-way interaction between time, education group, and age (at baseline) on brain system segregation, $F(1,183)=6.688$, $p=.010$, $CI_{95\%}[0.006, 0.045]$.

1.2 – Educational attainment does not moderate aging-accompanied changes in brain structure

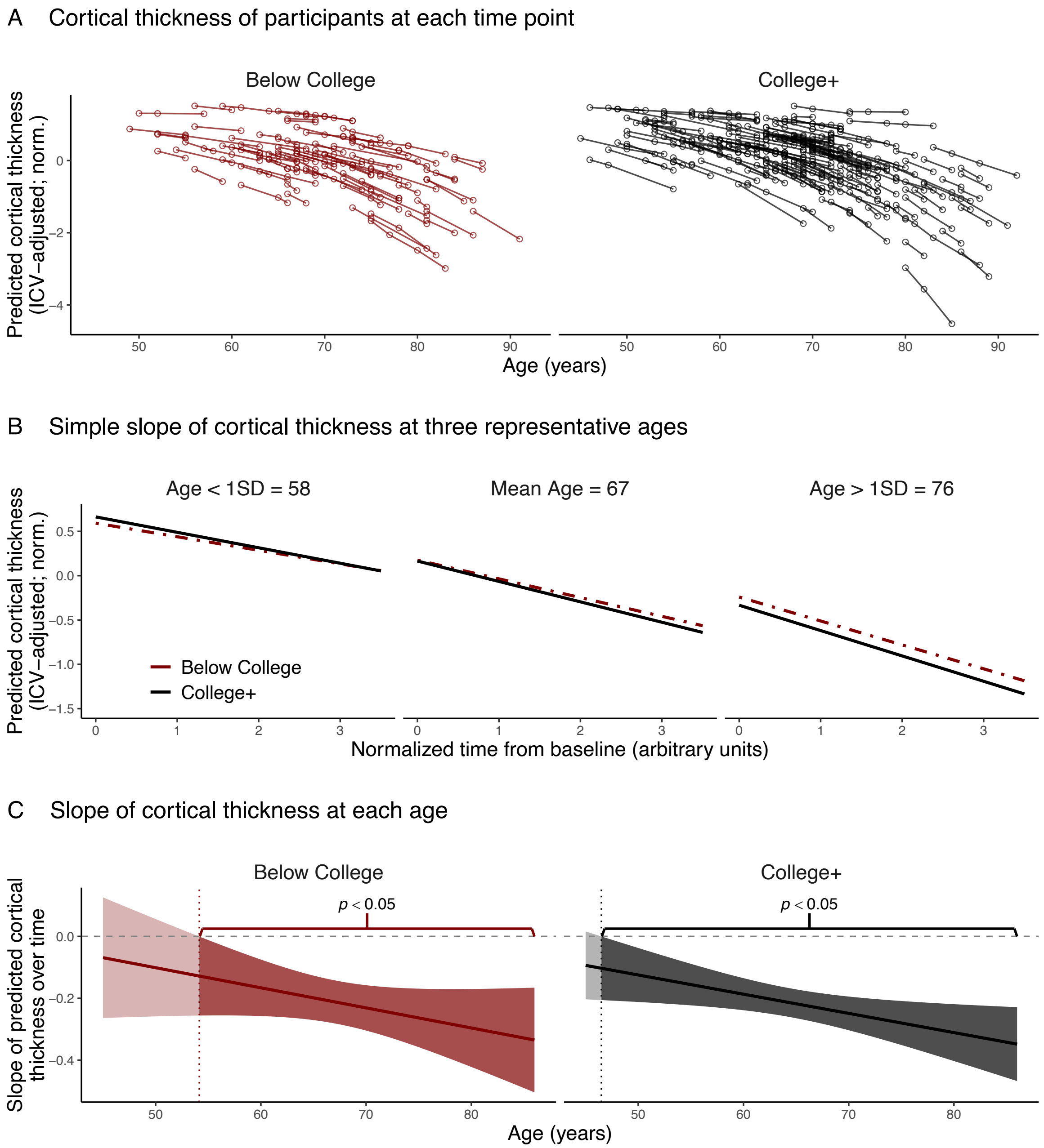

Figure S1 – Changes in cortical thickness are not moderated by education and age at baseline. The **(A)** changes in cortical thickness, **(B)** simple slope analysis, and **(C)** Johnson-Neyman analysis are plotted to illustrate that the linear mixed effects model predicting cortical thickness revealed no interaction between time, education group, and age at baseline ($F(1,178)=0.003$, $p=.954$, $CI_{95\%}[-0.005, 0.005]$). In **(C)**, the predicted slope calculated from the linear mixed effects model for each age within both education groups is illustrated; the envelopes represent the 95% confidence intervals. While there was an absence of a 3-way interaction, there was a statistically significant two-way interaction between time and age at baseline which revealed that cortical thinning was greater in older adult individuals, $F(1,173)=6.711$, $p=.010$, $CI_{95\%}[-0.011, -0.002]$. This pattern is evident by comparing the steeper negative slopes in the 76y vs. 58y panel in **(B),** and the increasingly negative slope values with increasing age as shown in the Johnson-Neyman plot (**C**).

1.3 – The relationship between education and changes in brain system segregation varies across different types of functional systems

In Figure 4 of the main text, it is apparent that there may exist distinctions in the segregation of different types of brain systems, as a function of educational attainment. Here, we tested this hypothesis. Different functional systems can be broadly categorized based on whether they are primarily involved with sensory-motor processing or in more associative processing (1, 2). Brain system segregation can be calculated for each of these subsets of brain systems independently. To do so we adapted our primary formula (i):

$$(i)\ Brain\ System\ Segregation = \frac{\frac{\sum_{w}^{W} Z_w}{W} - \frac{\sum_{b}^{B} Z_b}{B}}{\frac{\sum_{w}^{W} Z_w}{W}}$$

where Z is a Fisher's z-transformed correlation value, representing an edge between a pair of nodes. When calculating brain system segregation across the entire network, $Z_w$are the edges (correlation) between pairwise nodes that belong to the same system (within-system), $Z_b$are the edges between pairwise nodes that belong to different systems (between-system), W is the total number of within-system edges across all sub-networks, and B is the total number of between-system edges across all sub-networks. This is the usage for calculating brain system segregation that was described in the main manuscript. This formula was adapted for calculating brain system segregation for each of the two subsets of systems examined in the present report (i.e., association or sensory-motor) (ii):

$$(ii)\ Brain\ System\ Segregation\ (subset) = \frac{\frac{\sum_{w}^{W_t} Z_w}{W_t} - \frac{\sum_{b}^{B_t} Z_b}{B_t}}{\frac{\sum_{w}^{W_t} Z_w}{W_t}}$$

When calculating brain system segregation for each subset of systems, the aggregation of edges is limited to the brain systems that are part of the subset. Given a subset t (e.g., sensory-motor), which includes multiple brain systems (i.e., visual, auditory, hand-somatosensory, mouth-somatosensory), Z_w are the edges (correlations) between pairwise nodes that belong to the same system (within-system) that are part of the subset t (e.g., the within system correlations of the 4 sensory-motor systems). W_t is the total number of within-system edges across all sub-networks that belong to subset t (e.g., W_t is the number of within-system edges across the 4 sensory-motor systems). $Z_b$are the edges between pairwise nodes that belong to different systems (between-system) where one of the node belongs to subset t (e.g., correlations between nodes from each of the 4 sensory-motor systems to all other systems of the brain network including sensory-motor systems to one another but also the sensory-motor systems to the association systems). B_t is the total number of between-system edges across all sub-networks that had one node belong to subset t.

The specific functional systems (i.e., sub-networks) used for examining changes in sensory-motor and association system segregation were as follows: sensory-motor (visual, hand somato-sensory, mouth somato-sensory, auditory); association (frontal-parietal control, ventral attention, dorsal attention, default mode, cingulo-opercular control, medial temporal parietal, salience, hippocampus, superior temporal, parietal memory, memory retrieval; see **Figure 2A** of main text).

In a linear mixed model analyzing whether system subset (i.e., association vs. sensory-motor) moderates the interaction between time, education group, and age at baseline on brain system segregation values, where system subset was modeled as a random effect to allow for different intercept and slope for segregation values from the different subsets, no significant 4-way interaction was found (F(1,173)=0.043, p=.835, $CI_{95\%}$[-0.006, 0.005]). Notably, the 3-way interaction predicting brain system segregation (regardless of system subsets) remained significant (time × education group × age at baseline), F(1,212)=6.699, p=.010, $CI_{95\%}$[-0.016, -0.002]. **Figure S2** visualizes the predicted values, slopes and Johnson-Neyman plots of the two types of systems, in which both resembles the pattern observed for whole-brain system segregation. For example, in the Johnson Neyman plots (**Fig–S2C**) that illustrate the time × education group × age interaction, both association and sensory-motor systems exhibit a similar pattern (age-related decrease in slope among Below College, and slope hovering around 0 in College+).

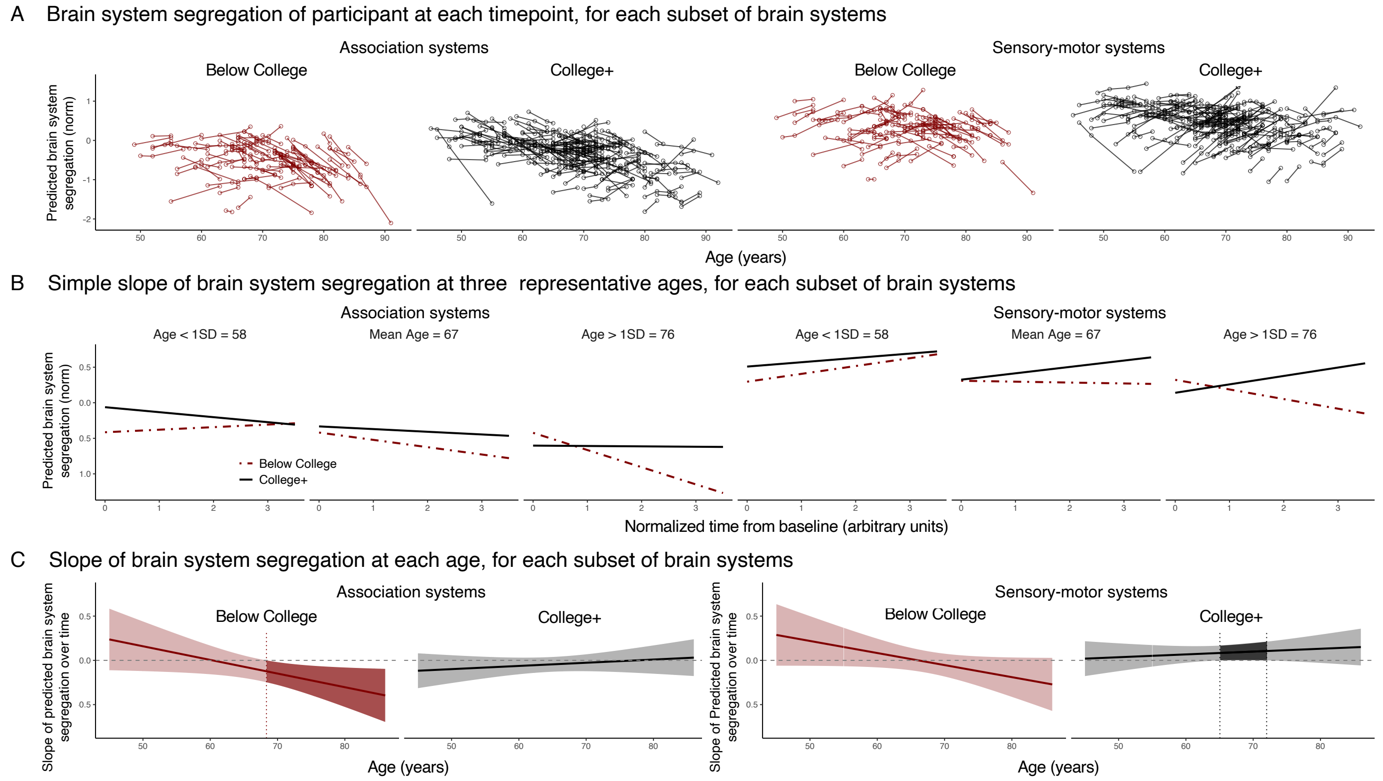

Figure S2 –Association and sensory-motor systems exhibit education-related differences in age-accompanied decline in brain system segregation. (A) Predicted brain system segregation value for association systems (left) and sensory-motor systems (right) from the linear mixed-effects model plotted against a participant's age when the brain scan was acquired. **(B)** Simple slope analysis depicts the predicted slope of brain system segregation of association systems (left) and sensory-motor systems (right) at three representative ages: 58, 67 and 76 (age < 1SD, mean age, and age > 1SD across the entire sample, respectively). Brain system segregation declines in older participants without a college degree (dashed red line in middle and right panel [age=67 & 76]), but stays relatively flat for individuals with a college degree regardless of age (solid black line). **(C)** A Johnson-Neyman analysis fully illustrates how changes in brain system segregation over time (y-axis) differs based on the participants' age (x-axis) and educational attainment. The predicted slopes calculated from the linear mixed effects model for each age within both education group is illustrated; envelopes represent the 95% confidence intervals. In participants without a college degree (left panel), the analysis identifies the ages at which the longitudinal changes in brain system segregation are statistically significant (darker shade representing a slope with $p < .05$, where 95% interval of the slope does not cross 0). While the patterns are similar, significant changes in older age Below College adults are statistically significant in association systems but do not reach statistical significance in sensory-motor systems.

1.4 – Brain system segregation decline predicts impending cognitive and functional impairment

1.4.1 – Changes in global CDR are predicted by changes in brain system segregation

While clinical dementia rating sum of boxes score (CDR-SB) offers better differentiation of cognitive impairment, the global cognitive dementia rating (CDR) score (which is derived from the CDR-SB) is the more familiar and widely used score among clinicians and researchers. For reference, we repeated the analyses conducted on CDR-SB by replacing the dependent variable with CDR, where time, age at baseline, change in brain system segregation, APOE status, and AD-related pathology were the predictors, while controlling for the following covariates: gender, average head motion, days between baseline and last MRI scan, and education group. The three-way time × age at baseline × change in brain system segregation interaction remained significant, $F(1,254)=8.182$, $p=.005$, $CI_{95\%}[-0.009, -0.002]$. The time × age at baseline × AD-related pathology interaction remained marginally significant, $F(1,236)=3.113$, $p=.079$, $CI_{95\%}[-0.007, 0.000]$, whereas the interaction between time, age at baseline, and APOE status was further attenuated, $F(1,237)=2.568$, $p=.110$, $CI_{95\%}[-0.006, 0.001]$. Similar to the model predicting CDR-SB, none of the higher order interactions were significant, $F\text{s}<1.969$, $p\text{s}>.162$.

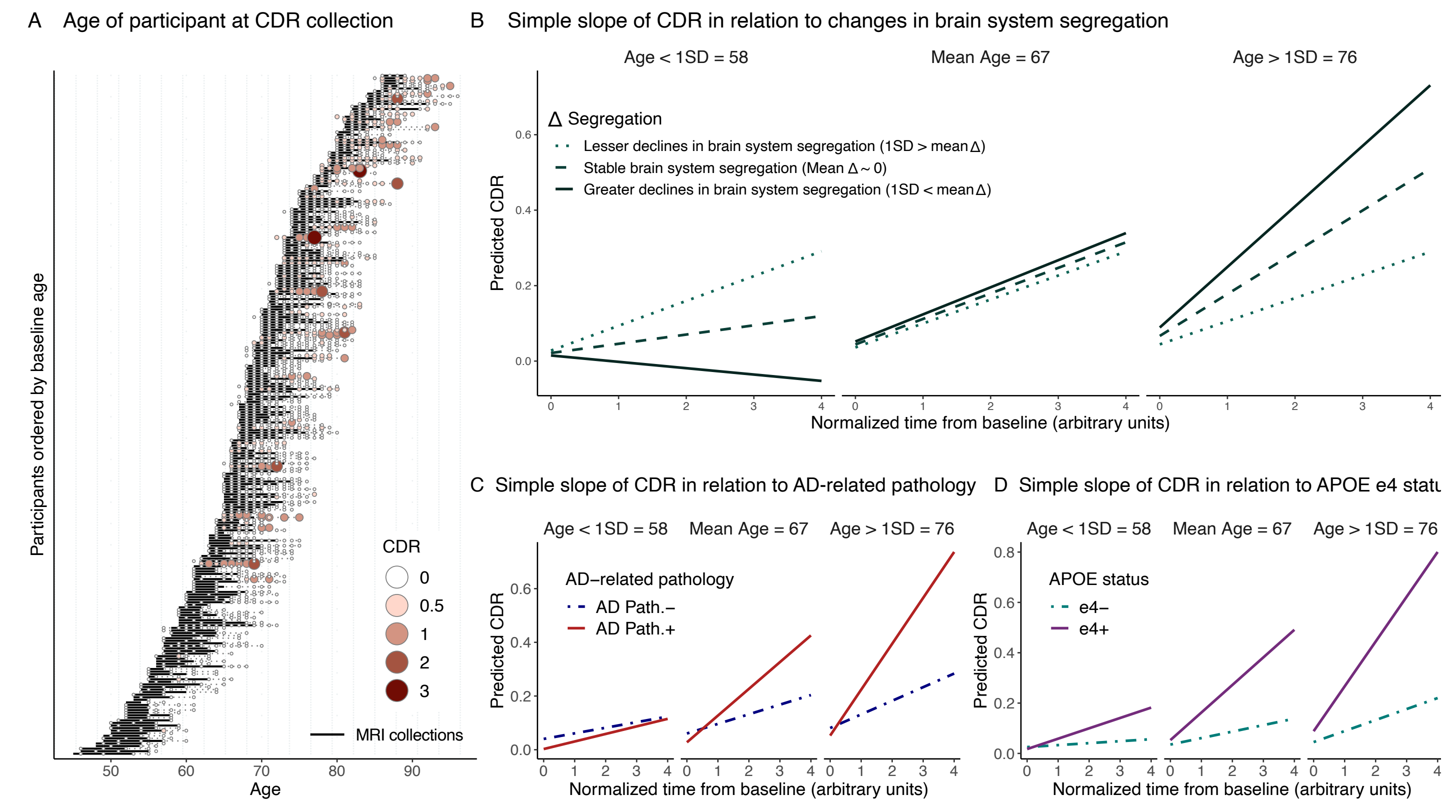

Figure S3 – Change in global CDR is predicted by changes in brain system segregation, independent of AD-related brain pathology and APOE e4 status. (A) Participant's clinical sessions (CDR data collection) that were collected within 1 year of baseline MRI (resting-state) scan and any time after (including sessions after their last MRI scans) were included. 96% of participants had available 3-17 clinical sessions. The size and color of the circles represent global CDR (larger size and darker red represent higher CDR). The dark lines represent the period of time between baseline and last MRI scan for each participant. **(B/C/D)** Simple slope plots depict the predicted slope of CDR at three representative ages: 58, 67 and 76. **(B)** Greater declines in brain system segregation (solid line) are associated with increasing CDR in older adults. Similar patterns, although statistically not significant in the present sample (see text), are observed for **(C)** presence of baseline AD-related pathology (aggregate category based on the presence of elevated CSF pTau and/or elevated cortical amyloid; solid line indicates the presence of pathology) and **(D)** positive APOE e4 carrier status (solid line indicates presence of AD genetic risk), separately.

1.4.2 – Further examination of the temporal relationships between brain network and clinical changes

As noted in the main text, a linear mixed-effects model revealed that changes in brain system segregation interact with time and age at baseline to predict changes in CDR-SB. This analysis used all clinical data collected after baseline MRI, including those collected after the last MRI scan,. In addition, AD-related pathology and APOE status both exhibited marginal interactions with time and age at baseline. The analysis included CDR-SB data collected after the last MRI scan for the majority of participants. Here, including only the CDR-SB data observed in between the baseline and last MRI session as a dependent measure also reveals a significant three-way interaction: time × age at baseline × change in brain system segregation, $F(1,248)=5.430$, $p=.021$, $CI_{95\%}[-0.040, -0.003]$. As highlighted in the main text however, changes in brain system segregation predict impending changes in CDR-SB even while controlling for the CDR-SB changes that were coincident with the MRI scans.

1.5 – Changes in brain system segregation may exhibit additive effects with both APOE status and baseline AD-related pathology to predict long-term changes in CDR-SB

As detailed in the main text, there was an absence of a 5-way interaction between time, age at baseline, change in brain system segregation, APOE status and baseline AD-related pathology on CDR-SB ($F(1,241)=0.002$, $p=.961$, $CI_{95\%}[-0.022, 0.021]$). In addition, there were no significant 4-way interactions (Fs<2.608, ps>.108). Given the number of terms in the model, the present study may be under-powered to detect these higher-order effects. However, based on the assumption that there may be additive consequences of exhibiting these biomarkers, here we depict the relationships between time, age at baseline, change in brain system segregation, APOE status and AD-related pathology. As can be seen in **Figure S4**, among the oldest adults, having 2 out of 3 of the measures (APOE e4+, presence of AD-related pathology [aggregated category based on the presence of elevated CSF pTau (> 67 pg/mL) and/or elevated cortical amyloid (>1.42 SUVR)] at baseline, or decreasing brain system segregation) is associated with increasing dementia severity (increased CDR-SB). To this end, the greatest cognitive decline is observed among older individuals who exhibit all three putative risk markers (APOE e4+, presence of AD-related pathology at baseline, and greater amounts of declining brain system segregation; see dark green line in bottom right panel). Finally, the model does not associate worse cognitive decline with greater brain system segregation decline among participants that are APOE e4- and have no AD-related pathology (top row).

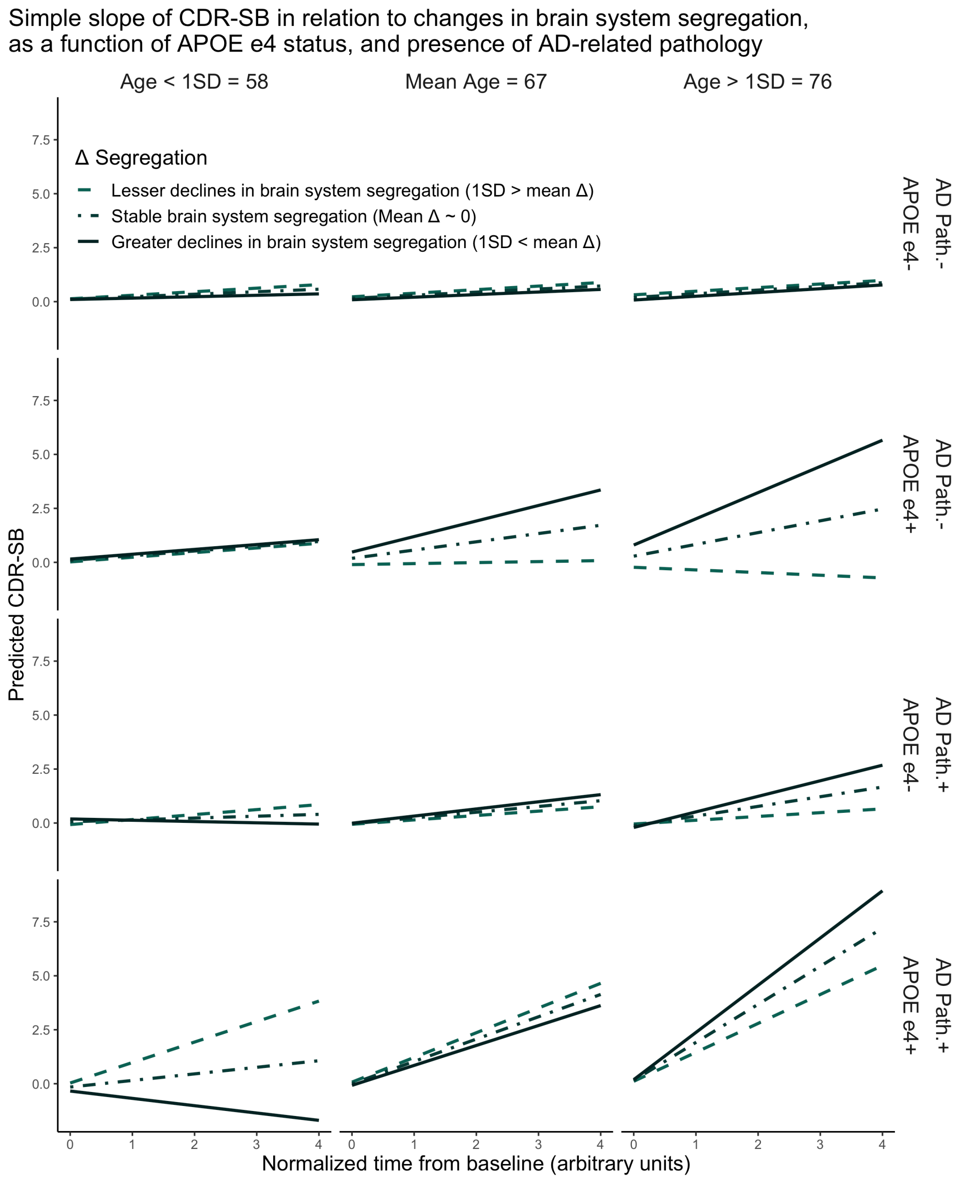

Figure S4 – Possible additive effect of change in brain system segregation, baseline AD-related pathology and APOE status on longitudinal changes in dementia severity. The simple slope plots illustrating predicted CDR-SB (dementia severity) change over time across participants at three representative ages (58, 67, 76), separated by their changes in brain system segregation (colored lines), APOE status, and AD-related pathology (each row is a combination of APOE status by AD-related pathology). Participants with at least one copy of the APOE e4 allele were categorized as APOE e4+. AD-related pathology is an aggregated category based on the presence of elevated CSF pTau and/or elevated cortical amyloid measured at participant's baseline scan (participants with one or more elevated AD-related pathology marker were categorized as positive for AD-related pathology). In older adults with either genetic risk (e4+; 2nd row) or AD-related pathology (AD Path.+; 3rd row), having greater decline in brain system segregation (solid line) is associated with greater change in CDR-SB (steepest slopes). Consistent with this, in older adults with both genetic risk and AD-related pathology (e4+ & AD Path.+; 4th row, rightmost panel), the model predicts that a substantial amount of increases in CDR-SB (i.e., worsening cognition) will occur across all levels of brain system segregation change. Again however, there is a dose-response effect in relation to declines in brain system segregation, whereby greater brain system segregation declines are associated with greater cognitive decline.

1.6 – The relationship between educational attainment and changes in brain system segregation persists while controlling for additional environment-related variables

Educational attainment is considered to be relevant to variability in an individual's environment. In Figure 1 of the main text, we included other SES-related information that are available for a subset of the cohort, to illustrate the relationship between educational level and other environment variables. Using that subset of participants (n=212) that have available socioeconomic index (SEI) and area deprivation index (ADI), we controlled for SEI and ADI while examining the 3-way interaction between time, education group, and age at baseline on brain system segregation. The model remained significant in this sub-sample: $F(1,150)=7.211$, $p=0.008$, $CI_{95\%}[-0.024, -0.004]$, demonstrating education relationships with changes in brain system segregation are also evident even while controlling for other available environment-related variables. We note that ADI includes household income in its estimation, thus median household income was not simultaneously added to the model.

1.7 – Examination of relationships across data parameters (amount of resting-state data per participant) and graph constructions (age-specific brain area parcellations)

Beyond processing steps described throughout the main text, additional considerations were made with respect to potential age-related differences in (i) resting-state data frames retained due to head motion correction (3) and (ii) topography of cortical areas which would have relevance for nodes in brain network construction (4). Collectively, supplemental analyses yielded qualitative similar results across ranges of data frame thresholds and different set of nodes, providing evidence that the observed results were not specific to certain choices in preprocessing parameters or node sets.

1.7.1 – Impact of varying amount of resting-state data per participant

Increasing age is associated with increasing in-scanner head movement (5). Head motion systematically alters resting-state correlations (6); in order to minimize this source of non-neuronal noise, data 'scrubbing' procedures were employed to flag and remove high movement frames. While scrubbing allows each participant to be 'cleaner' in terms of the quality of the resting-state data, participants (and scans) can have differing amounts of lower-motion useable frames, where every scan for a given participant may have varying amounts of head motion that surpass the frame-displacement threshold used for framewise 'scrubbing'**.** Further, including a different number of frames contributing to a within participant longitudinal analysis might result in differential power to detect measures of interest for each participant and timepoint, which might bias results. Thus, it is critical to use an equivalent number of frames across both participants and sessions, and the processing procedure requires having to decide the amount of data to examine per scan. As such, there is an inherent tradeoff that must be balanced, between number of frames and number of participants included in the analysis, whereby increasing the number of frames per participant scan decreases the total participant sample size (n=232 at 125 frames for all longitudinal scans; n=208 at 150 frames for all longitudinal scans; n=151 at 200 frames for all longitudinal scans).

Our goal was to apply adequate motion correction while also both maximizing and equating the available amount of data per subject and sessions. In the analyses presented in the main text, all resting-state scans consisted of 100 frames (i.e., every session of every participant). Within the sampled frame-number/subject/session parameter-space, the primary interactions between time, education group, and age at baseline remain significant for brain network system segregation (125 frames: $F(1,158)=7.854$, $p=.006$, $CI_{95\%}[-0.024, -0.004]$; 150 frames: $F(1,139)=4.462$, $p=.036$, $CI_{95\%}[-0.021, -0.001]$). Consistent with the tradeoffs discussed above, further increasing the number of required frames to 200 results in the loss of a significant number of participants that meets the requirement (n=151 at 200 frames vs. n=208 at 150 frames; ~27% decrease in sample size). In keeping with this, using 200 frames, the three-way interaction between time, education group, and age at baseline on brain system segregation was no longer statistically significant, $F(1,109)=1.663$, $p=.200$, $CI_{95\%}[-0.021, 0.004]$. Additionally, after controlling for baseline covariates (AD-related

pathology, clinical status, APOE status, cardiovascular health, depressive symptom, traumatic brain incidents), demographics (race, sex), and head motion, the three-way interaction is significant at 125 frames ($F(1,145)=5.848$, $p=.017$, $CI_{95\%}[-0.024, -0.002]$), but attenuates at 150F and 200F ($F_{150F}(1,130)=2.942$, $p=.089$, $CI_{95\%}[-0.021, 0.001]$; $F_{200F}(1,100)=1.228$, $p=.270$, $CI_{95\%}[-0.022, 0.006]$). Similarly, controlling for longitudinal brain structure (cortical thickness), the three-way interaction also remained significant at 125 frames ($F(1,161)=6.796$, $p=.010$, $CI_{95\%}[-0.023, -0.003]$), but attenuates at 150F and 200F ($F_{150F}(1,143)=3.634$, $p=.059$, $CI_{95\%}[-0.020, 0.000]$; $F_{200F}(1,111)=1.413$, $p=.237$, $CI_{95\%}[-0.020, 0.005]$).

1.7.2 – Age-group specific brain area parcellations to generate brain network nodes

The focus of the presented work was on constructing brain graphs that were derived from spatially consistent brain area parcellations across middle-aged and older participants. In addition, a surface-based node set with uniform nodes surrounding putative areal centers was chosen to (i) mitigate partial-volume effects that affects older adults with atrophy, and (ii) avoid including signals from areas that are more likely to differ across participants (i.e., putative areal borders; 7). Furthermore, a consistent set of community assignment for the brain nodes was used across all participants (3). While this approach ensures that the construction of brain graphs across participants of varying age is consistent, it does not account for any possible age-related differences in brain area topography (4).

Here, analyses in the main manuscript are repeated using age-group specific node sets that respect possible age-related differences in brain area topography and functional system identity (i.e., community assignment; 4). To summarize, the results are qualitatively similar to analyses using the primary node set in the main manuscript, providing evidence that the reported results are not biased by any potential age-related differences in brain area topography.

Generating age-group specific 3mm-radius node sets from parcellations

In a previous study, five age-group specific resting-state functional correlation (RSFC) parcellations were generated using a watershed method on RSFC gradient maps (4). While the functional parcellations are largely consistent across the healthy adult lifespan, the study observed noticeable differences in the precise spatial topography of the parcels that may be attributed to age-related variation in brain structure (e.g., differences in cortical thickness and anatomical alignment). Accordingly, the following analyses were conducted using nodes based on age-group specific parcellation to account for subtle age-related differences in topography of the network nodes and functional system assignments. In the present study, participants' age ranged from 45 to 86 at baseline, which spanned four age-groups specific parcellations from Han et al.: Middle-Early = 35-49y; Middle-Late = 50-64y; Older-Early = 65-79y; Older-Late = 80y+.

To reduce uneven contribution across parcels of varying sizes, 3mm radius surface-based disks were constructed around the center of each parcel for each age group parcellation. The nodes were derived using

the following steps: (i) calculating the volumetric center by averaging the volumetric 3D coordinate of all vertices within a parcel in 32k fs_LR spherical space, and (ii) mapping the volumetric center onto the surface by identifying the surface vertex with the shorted Euclidean distance with the volumetric center, (iii) creating 3mm-radius surface disks around surface vertices identified in step (ii).

Han et al. (2018) created functional system assignments for parcels by conducting Infomap community detection (8) on each age-group specific parcellation across 3-10% edge densities (in steps of 0.1% from 3% to 5% and in steps of 1% from 5% to 10%). A consensus approach was used to label the detected community assignments based on a published functional system atlas (3). These community assignments were applied to the 3mm surface nodes generated here. A small portion of parcels were not assigned a final community label (e.g., parcels that belong to communities with fewer than 5 nodes). The unassigned nodes were excluded from the participants' brain network matrix before calculating brain system segregation (see **Fig. S5** for modified Han et al. nodes).

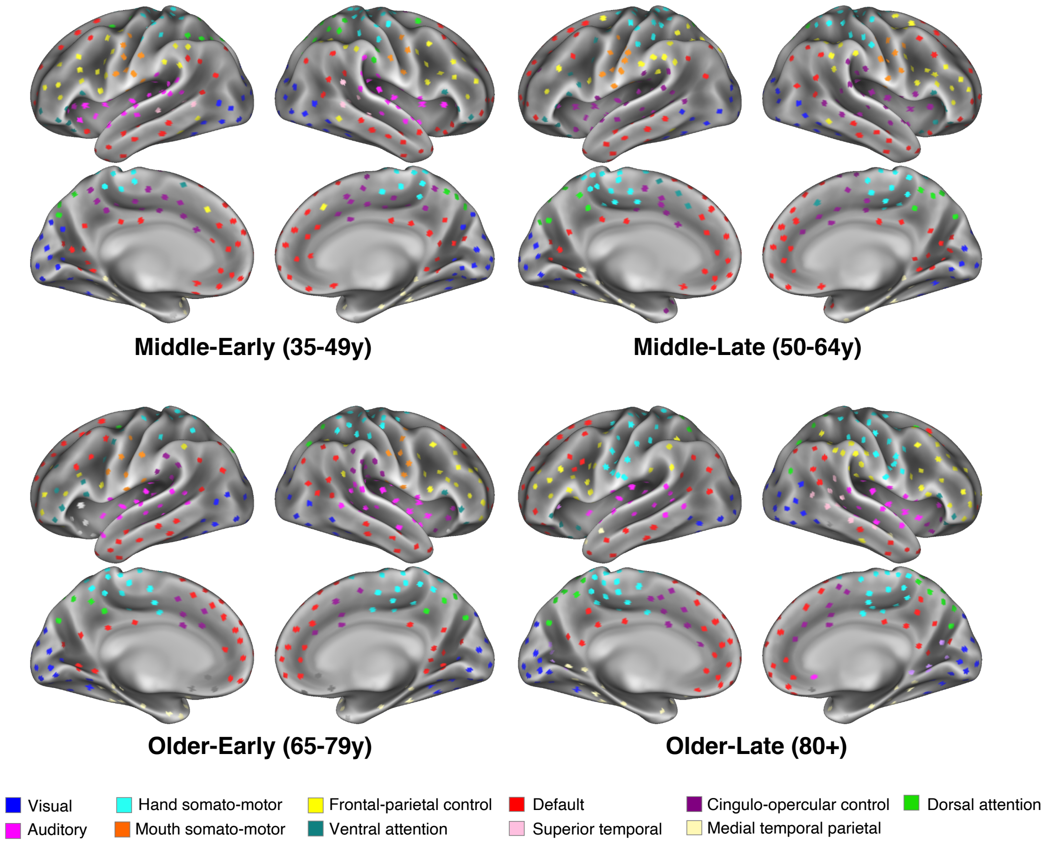

Figure S5 – Age-group specific nodes, modified from the Han et al. (2018) parcellation maps. Age-cohort specific parcellations were obtained from (Han et al., 2018), for four age-groups: Middle-Early = 35-49y; Middle-Late = 50-64y; Older-Early = 65-79y; Older-Late = 80y+. Participants' brain networks (for all scans) were constructed using the parcellation map corresponding to their age during the first (baseline) scan. To reduce uneven contribution across parcels of varying sizes, 3mm-radius surface-based disks (depicted) were constructed around the center of each parcel for each age group parcellation.

Results using age-group specific nodes

Each participant's brain network at each session was rebuilt using age-group specific nodes based on their age at the baseline scan. For example, if a participant was 64 at baseline and 67 at a subsequent timepoint, all brain networks of all of their sessions were constructed using the Middle-Late node set. Inclusion of age-group specific nodes yielded consistent results with the observations reported in the main manuscript. The three-way interaction between time, education group, and age at baseline is significant $F(1,180)=5.581$, $p=.019$, $CI_{95\%}[-0.020, -0.002]$. The three-way interaction attenuated to a marginal effect after controlling for all baseline covariates (clinical status, APOE status, cardiovascular health, depressive symptom, traumatic brain incidents), demographics (race, sex), and head motion, $F(1,162)=3.437$, $p=.066$, $CI_{95\%}[-0.019, 0.000]$, but remained significant after controlling longitudinal cortical thickness, $F(1,180)=4.993$, $p=.027$, $CI_{95\%}[-0.019, -0.001]$.

Similar to section 1.7.1, these models were re-examined using the modified Han et al. nodes at multiple frame-kept thresholds (125, 150, 200). The basic three-way interactions remained significant from 125 and 150 minimum frames ($F_{125F}(1,140)=6.376$, $p=.013$, $CI_{95\%}[-0.021, -0.003]$; $F_{150F}(1,130)=4.053$, $p=.046$, $CI_{95\%}[-0.020, -0.000]$). The participant sample size is significantly diminished at 200 frames (n=151) and the effect is attenuated to a marginal effect, $F(1,106)=3.466$, $p=.065$, $CI_{95\%}[-0.024, 0.000]$. Overall, the predicted decreases in brain system segregation in Below College older adults were verified using age-group specific area parcellation nodes.

1.8 – Education attainment distinctions are well captured by the Below College vs. College+ categorization

The completion of an undergraduate college degree in the United States has been linked to a considerable number of health and socioeconomic advantages (9). Accordingly, categorizing education based on this distinction allows one to capture meaningful opportunities associated with higher educational attainment. To provide an illustrative example, a 1-unit difference between 14 and 15 years of education (in both cases, some college) is quite different from the difference between 15 and 16 years of education (some college in the former, while typically completing college in the latter). These types of non-linear distinctions are not well captured by a continuous variable. **Figure S6** illustrates that the proportion of participants with different years of education aligns with the estimated degree-completion years for high school (12 educational years) and college (16 educational years and above) in the United States**.**

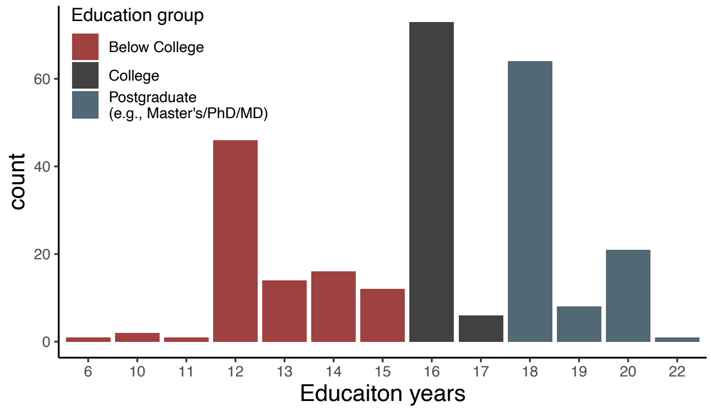

Figure S6 – A large proportion of participants have education years that align with estimated degree completion. Participant reported education years are plotted as a histogram, showing the peaks at 12y, 16y, and 18y, aligning with typical degree-completion years in the United States (i.e., 12y=high school, 16y=college, 18y=master's). Bars are colored based on how education years can be binned into degree-completion categories. There is also a less prominent peak at 20y, which could correspond to doctorate degrees.

As seen in **Figure S6**, it is possible that the distinction between individuals with a college degree might be further differentiated from those with a graduate degree in the present sample. Importantly, this is an inference as data on degree completion was not available for this study sample. When education is examined with 3 groups (separated by below college [6-15 years], college completion [16-17 years], and postgraduate degree completion [18 years+]), brain system segregation is also significantly predicted by a 3-way interaction between time, education group, and age at baseline, $F(2,177)=4.341$, $p=.014$, $CI_{95\%}[0.004, 0.025]$. As shown in **Figure S7**, the interaction is driven by Below College older adults exhibiting a notably different pattern of longitudinal changes in brain system segregation compared to College and Postgraduate older adults.

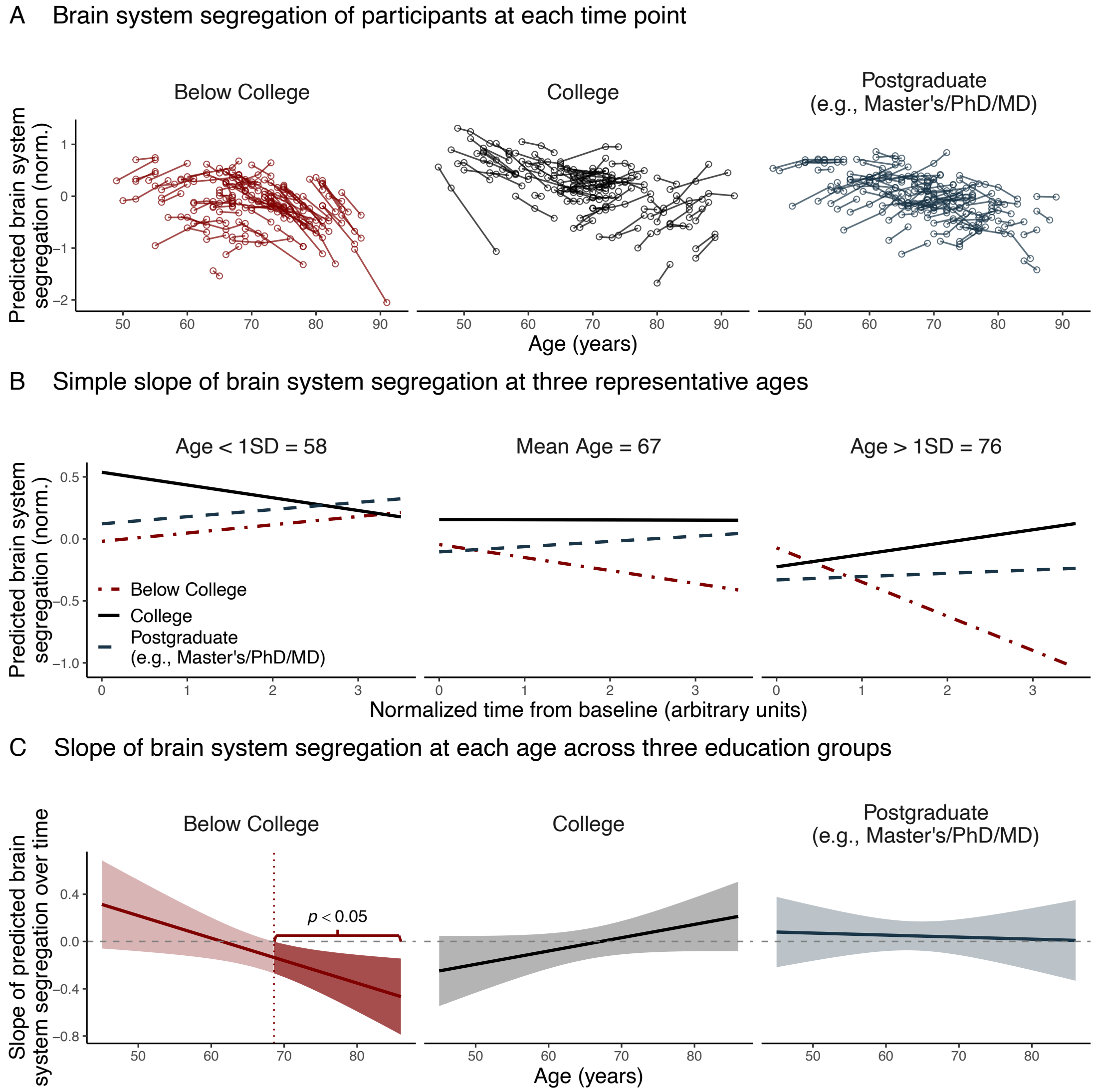

Figure S7 – Brain system segregation declines most in older adults without a college degree, when compared with individuals with college or postgraduate degrees. (A) Predicted brain system segregation value from the linear mixed-effects model plotted against a participant's age when the brain scan was acquired. Greater decline in brain system segregation is evident in participants who are older (~65y+) and without a college degree, compared to more educated individuals (both individuals with college or postgraduate degrees). **(B)** Simple slope analysis depicts the predicted slope of brain system segregation at three representative ages: 58, 67 and 76 (age < 1SD, mean age, and age > 1SD across the entire sample, respectively). Brain system segregation declines in older participants without a college degree (dashed red line in middle and right panel [age=67 & 76]), but stays relatively flat for individuals with a college or postgraduate degree regardless of age (solid black line). **(C)** A Johnson-Neyman analysis fully illustrates how changes in brain system segregation over time (y-axis) differs based on the participants' age (x-axis) and educational attainment. The predicted slope calculated from the linear mixed effects model for each age within all education groups are illustrated; the envelopes represent the 95% confidence interval. In participants without a college degree (left panel), the analysis identifies the ages at which the longitudinal changes in brain system segregation are statistically significant (darker shade representing a slope with $p < .05$, where 95% interval of the slope does not cross 0). The decline in brain system segregation is statistically significant for individuals without a college education starting at approximately 69y; reliable changes of brain system segregation are not evident at any segment of age for those with a college degree (middle panel) or postgraduate degree (right panel). To facilitate the comparison between education groups, the slope of predicted brain system segregation for all three education groups are shown for the full age-range of the entire sample (45-86y).

In keeping with our statements regarding non-linear impacts of educational attainment, treating education as a continuous variable reveals a marginal 3-way interaction in predicting changes in brain system segregation at $F(1,238)=2.980$, $p=.086$, $CI_{95\%}[-0.001, 0.017]$. But as we have elaborated above; we did not hypothesize the education effect to be linear in a continuous fashion. Rather, and as illustrated throughout this report, it is likely that in the context of enabling an individual to access additional resources and opportunities, the distinction of degree completion between College+ and Below College is most appropriate.

1.9 – Distribution of CDR-SB over time across both education groups

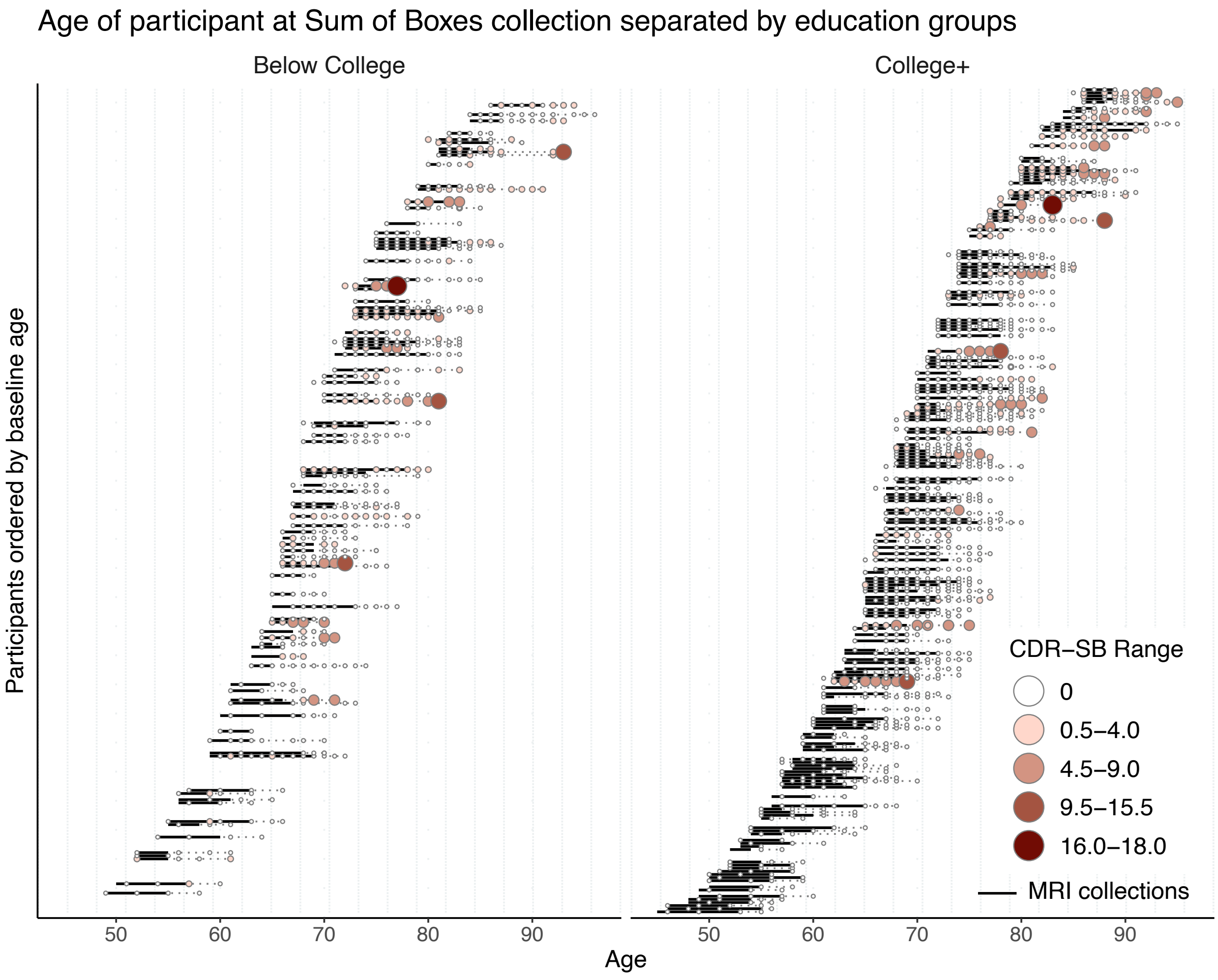

Figure S8 - Distribution of CDR-SB over time across both education groups.

2. SUPPLEMENTAL REFERENCES

1. Mesulam MM (1990) Large-scale neurocognitive networks and distributed processing for attention, language, and memory. *Ann Neurol* 28(5):597-613.
2. Posner MI & Petersen SE (1990) The attention system of the human brain. *Annual review of neuroscience* 13:25-42.
3. Power JD*, et al.* (2011) Functional network organization of the human brain. *Neuron* 72(4):665-678.
4. Han L*, et al.* (2018) Functional Parcellation of the Cerebral Cortex Across the Human Adult Lifespan. *Cereb Cortex* 28(12):4403-4423.
5. Savalia NK*, et al.* (2017) Motion-related artifacts in structural brain images revealed with independent estimates of in-scanner head motion. *Human brain mapping* 38(1):472-492.
6. Power JD, Barnes KA, Snyder AZ, Schlaggar BL, & Petersen SE (2012) Spurious but systematic correlations in functional connectivity MRI networks arise from subject motion. *NeuroImage* 59(3):2142-2154.
7. Chan MY, Park DC, Savalia NK, Petersen SE, & Wig GS (2014) Decreased segregation of brain systems across the healthy adult lifespan. *Proc Nat Acad Sci USA* 111(46):E4997-5006.
8. Rosvall M & Bergstrom CT (2008) Maps of random walks on complex networks reveal community structure. *Proceedings of the National Academy of Sciences of the United States of America* 105(4):1118-1123.
9. Abel JR & Deitz R (2014) Do the benefits of college still outweigh the costs? *Current Issues In Economics and Finance* 20(3):9.
